# Leveraging Meteorological Reanalysis Models to Characterize Wintertime Cold Air Pool Events Across the Western United States from 2000 to 2022

**DOI:** 10.3390/atmos16121325

**Published:** 2025-11-24

**Authors:** Jacob Boomsma, Heather A. Holmes

**Affiliations:** 1 Department of Atmospheric Sciences, University of Utah, Salt Lake City, UT 84112, USA; 2 Department of Chemical Engineering, University of Utah, Salt Lake City, UT 84112, USA

**Keywords:** stable stratification, temperature inversions, mountain meteorology, radiosonde, ERA, wintertime air pollution

## Abstract

Wintertime cold air pools (CAPs) are common across the Western United States and result in cold, dense air trapped in valley basins. The CAPs are characterized by a stable atmospheric boundary layer, leading to cold air and low wind speeds. While CAP formation occurs nightly, the CAP conditions can persist into daytime and often last for multiple days (i.e., persistent cold air pool or PCAP), resulting in poor air quality in populated areas. The presence and strength of CAPs can be calculated using data from radiosondes, surface weather stations at varying elevations, and indirectly through air pollution monitors. Because vertical profile data are often limited to twice daily radiosondes, and are spatially sparse, numerical models can be a useful substitute. This work uses the European Centre for Medium-Range Weather Forecasts (ECMWFs) Reanalysis v5 (ERA) atmospheric reanalysis to provide data to classify wintertime CAP events without radiosonde observations. An automated CAP classification method using ERA outputs is evaluated using afternoon radiosonde observations in six cities (Salt Lake City, Utah; Reno, Nevada; Boise, Idaho; Denver, Colorado; Las Vegas, Nevada; Medford, and Oregon). Using this CAP determination method, days with CAP events are analyzed in 13 locations, 6 with radiosonde observations and 7 without, including the Central valley of California. The CAP classification method is evaluated at these 13 locations across the Western US over the study period of 2000–2022. The results show that the ERA model performs similarly to the radiosonde observations when used to identify CAP events. Therefore, ERA can be used to provide a reasonable estimate of CAP conditions when radiosonde data are unavailable. Providing consistent CAP classifications across space and time are necessary for regional scale CAP studies, such as human health effects modeling over large spatial and temporal scales.

## 1. Introduction

During the winter, mountain valleys often experience stable atmospheric boundary layers (ABLs) for multiple days, which can lead to poor air quality [1–6]. Most major cities in the western U.S. are in mountain valleys, which are susceptible to cold air pool (CAP) formation [6,7]. CAPs are associated with stable atmospheric boundary layers, with decreased mixing, lower boundary layer heights, and pollutant accumulation [4]. During multi-day CAP events, or persistent cold air pools (PCAPs), the PM_2.5_ concentrations increase and can exceed the 24 h PM_2.5_ National Ambient Air Quality Standard (NAAQS) of 35 μg m^−3^ [1–6], leading to adverse health outcomes. For example, in northern Utah, exposure to elevated air pollution concentrations are associated with a greater risk of cardiorespiratory health outcomes [8,9]. Because of the poor air quality during CAP events, straightforward methods of determining the existence and strength of a CAP are critical.

To characterize CAPs the atmospheric stability must be determined. The most common approach to determine atmospheric stability is to use the vertical temperature profile. On a plot of temperature versus height, small negative temperature gradients or positive temperature gradients (indicating a temperature inversion) indicate a stable atmosphere, while large negative temperature gradients indicate an unstable atmosphere. The adiabatic lapse rates for dry and saturated air, dry adiabatic lapse rate (DALR) and moist adiabatic lapse rate (MALR), are used to compare with the environmental lapse rate to determine atmospheric stability. The MALR is less than the DALR because of the moisture in the air that condenses as the air rises and cools, releasing latent heat. The DALR is a constant value, while the MALR is variable and depends on temperature and pressure. An environmental lapse rate greater than the DALR is considered unstable. While an environmental temperature profile that has a lapse rate less than the MALR is considered stable. An environmental lapse rate between the DALR and MALR is considered conditionally unstable because it depends on whether or not the parcel is saturated. Because potential temperature is a conserved quantity for a dry adiabatic process, it is often used to characterize atmospheric stability for unsaturated layers. If potential temperature decreases with height, an unsaturated layer is unstable while if potential temperature is constant with height, an unsaturated parcel would experience no acceleration when displaced vertically (i.e., neutral atmospheric stability). When potential temperature increases with height, stability depends on both the rate at which potential temperature increases through the layer and the layer’s water vapor concentration. While this type of analysis provides a method to characterize stability it has two major limitations (1) it is labor intensive and not automated and (2) it does not provide a quantitative measure of the atmospheric stability.

Previous studies use a bulk measure of atmospheric stability within a valley, known as the valley heat deficit (*VHD*), to classify CAPs [2,6,10]. The *VHD* method is widely used because it only requires a vertical potential temperature profile, which can be obtained from both observations and numerical models. Observations of vertical meteorological observations are sparse across the western U.S., but numerical models can be used instead. Examples of previous models that have been used to investigate CAPs, are the Met Unified Model (MetUM) [11], North American Regional Reanalysis (NARR) [7], and Weather Research and Forecasting Model (WRF) [12–14]. Leveraging model outputs to classify CAPs extends the regions where wintertime air pollution investigations can be performed (e.g., Central Valley of California).

In this study, we developed a method, using previously published methods as a starting point, that determines the existence and strength of a daytime CAP (i.e., afternoon 00Z temperature profile) in any location where numerical model outputs are available. With the overarching goal of numerically automating the CAP classification process for use with large datasets (e.g., >25,000 vertical profiles). The numerical CAP classification method is applied to vertical atmospheric profiles from model outputs in 13 locations across the western U.S. over 22 winters. In the six locations with radiosonde observations, the differences in CAP strength, temperature, and wind speed from observations and model outputs are compared to evaluate the effectiveness of using gridded model outputs to classify CAPs. Using the numerical CAP classification method, the number of CAP and PCAP days for each winter over the 22-year study period is summarized. Ultimately, the CAP classification results will be used to support a health effects study investigating the association between daily cardiorespiratory health outcomes and wintertime air pollution in the western U.S.

## 2. Materials and Methods

This study focuses on a 22-year winter period from winter 2000/2001 to 2021/2022, where winter is defined as 15 November to 15 February of each year. The 13 locations are shown in Figure 1 and include Salt Lake City, Utah; Reno, Nevada; Boise, Idaho; Denver, Colorado; Las Vegas, Nevada; Medford, Oregon; Ogden, Utah; Provo, Utah; Bakersfield, California; Fresno, California; Modesto, California; Sacramento, California; and Visalia, California. The basin characteristics, including elevation and mean ridge heights, for each location are provided in Table 1. Using observations and model results in each of these locations, over the 22-year period, CAP days are determined with the methods described below.

### 2.1. Radiosonde, Surface Meteorological Station, and Meteorological Reanalysis

Observational radiosonde measurements are taken twice daily (00Z and 12Z) in six of the cities: Boise, Denver, Las Vegas, Medford, Reno, and Salt Lake City with data available from the University of Wyoming [15]. Because stable ABLs are often evident in morning soundings, only afternoon radiosonde observations (00Z) are used to identify CAPs persisting longer than a diurnal cycle in this study. The radiosonde variables for each vertical profile include temperature, dew point temperature, elevation, pressure, wind speed, and wind direction. If any data is missing between 500 hPa and the surface, the profile is not used, and the radiosonde observation is considered missing. Additionally, radiosonde soundings are not always launched from the valley floor, which is the case in Reno where there is a 145 m difference between the valley floor and the radiosonde location. This discrepancy results in unclassified CAP events because the surface inversions can be missed or underrepresented (e.g., Colgan et al. (2021) [6]). To reduce this discrepancy, the observations from the surface meteorological station on the valley floor are appended to the radiosonde vertical profile in Reno using the method from Colgan et al. (2021) [6].

Automated surface observing system (ASOS) surface weather stations were used to provide data for the surface meteorological conditions in each study location. The ASOS are maintained by federal agencies and the data were accessed from MesoWest [16]. In locations with radiosondes, the ASOS site collocated with the radiosonde launch site was used, with the exception of Denver and Reno. In Denver, there are several ASOS stations near the radiosonde launch; KDEN was chosen because it is at the lowest elevation and provides data representative of the valley floor (21 km from the radiosonde site). In Reno, KRNO was chosen because it is the lowest elevation in the valley, located 7 km away from the radiosonde site. In locations without radiosonde observations, the nearest ASOS station to the city center was selected.

To obtain vertical profile data in locations without radiosonde observations (squares in Figure 1) ECMWF Reanalysis 5 (ERA) [17] is used. The ERA model domain is available worldwide at a relatively fine horizontal spatial resolution of 0.25 degrees, or roughly 31 km, from 1979 to the present. Because ERA has a coarser horizontal resolution than other models, and some valleys in this study are relatively small (Table 1), the ERA grid cell selection for each location is not trivial. The grid cell selection for each city is determined by the location of the ASOS surface meteorological station. For each station, the closest four grid cells are investigated to determine which grid cell best represents the surface conditions. The grid cell with the closest elevation to the ASOS station is selected, even if it is not the grid cell containing the surface weather station.

Similarly to how the surface observations are used with the radiosonde data to correct for elevation discrepancies, they are also used here with the ERA model output to improve the vertical temperature profiles. Because numerical models often struggle with capturing surface-based inversions [14,18], using surface temperature and wind observations instead of results from the surface model grid is expected to improve the CAPs classification on days with surface inversions. This hypothesis was tested in this study, and datasets labeled ERA_Adj_ replace the surface point of ERA with the ASOS surface observation. It is important to consider the timing of both the surface observation and radiosonde launch to match the proper observations. For example, the radiosonde is launched one hour before the radiosonde time stamp (2300Z for the 00Z radiosonde), and the time stamp on the surface station observations indicates the end of the 5 min sampling period (00Z sampling starts at 2255Z).

### 2.2. Creating a CAP Evaluation Test Dataset

To test the effectiveness of the proposed CAP method described below in Section 2.3, a qualitative CAP classification dataset is generated based on human detection of CAPs on individually plotted radiosonde data. Specifically, CAPs are determined by visual inspection of the radiosonde profiles to find stable layers associated with CAPs. The stable layer must be present between the surface and 1.5 times the mean ridge height for it to be classified as a CAP. The thickness of the stable layer is not considered. The stable layers and CAPs are determined by visually inspecting the afternoon sounding profiles on skew-T log-P diagrams, specifically examining temperature (lapse rate) and wind speed profiles. Any lapse rate more stable than moist adiabatic is considered stable. Because storms can have a vertical temperature profile that follows the moist adiabatic lapse rate, surface wind speed is also considered in the CAP determination (e.g., storms with a stable layer would have high wind speeds). Afternoon profiles (00Z) are used exclusively because the focus is on CAP events that persist after the common occurrence of a nighttime CAP. If a layer’s lapse rate is less than the moist adiabatic lapse rate, then the layer is considered stable. If a stable layer is accompanied by surface wind speeds ≤ 4 m s^−1^, then it is assumed a CAP is not likely to be present. This surface wind speed criteria was chosen based on the method proposed in Colgan et al. (2021) [6] who used 3 m s^−1^ in their study based on the valley cold pool classification proposed in Yu et al. (2017) [7]. Our initial testing found that the 3 m s^−1^ threshold was too strict so we increased the surface wind speed threshold to 4 m s^−1^. Based on this visual inspection, a test evaluation dataset is created for three winters: 2010/2011, 2015/2016, and 2021/2022 in the cities with radiosondes. Each afternoon sounding was divided into ‘yes’, ‘no’, and ‘maybe’ CAP days. ‘Yes’ days meet the criteria of having a stable layer, discussed above, and also meet the surface wind speed restriction (*WS* ≤ 4 m s^−1^). The ‘maybe’ days are visually inspected as a CAP based on the temperature lapse rate but do not meet the surface wind speed criteria. All other cases are considered ‘no’ days. Using this qualitative yes/no/maybe CAP dataset, the quantitative method developed for CAP classification can be evaluated.

### 2.3. Automated CAP Classification Method

The numerical CAP classification method shown here leverages previous CAP classification approaches with some modifications. We start with methods from Whiteman et al. (2014) [2] and Colgan et al. (2021) [6] and then modify the approach, in part, so ERA model outputs can be used in place of radiosonde observations. These approaches rely on the valley heat deficit (*VHD*) to quantify atmospheric stability [2], shown in Equation (1) as

(1) $$VHD=c_{p}\int_{sfc}^{h}\rho (z)[\theta (h)-\theta (z)]dz.$$

where $c_{p}$ is the specific heat of air at constant pressure (1005 J kg^−1^ K^−1^), sfc is the surface elevation (m ASL), h is the integration height (m ASL), ρ is the air density (kg m^−3^), θ is the potential temperature (K), and z is altitude (m). Equation (1) differs from the *VHD* shown in Whiteman et al. (2014) [2] in that h is a variable integration height and not the mean ridge height. In this study, we determine a suitable integration height for each temperature profile instead of using a static value based on the mean ridge height. We adjust the *VHD* equation to use a variable integration height for h to include elevated stable layers associated with subsidence inversions that can occur near, or just above, the ridge height (e.g., Colgan et al. (2021) [6]). Next, we describe the four specific steps for our new CAP classification method.

#### Step 1: Determine VHD Integration Height

Using a variable integration height in the automated CAP classification method requires an algorithm for selecting the height (*h* in Equation (1)). First, the maximum vertical extent of the atmosphere considered is limited, starting from the surface up to 1.5 times the mean ridge height. This prevents the CAP classification algorithm from reaching the tropopause or any high elevation stable layers that are not within the planetary boundary layer (PBL).

Then, to find the integration height, the CAP classification algorithm searches for stable layers in the vertical layer from the surface up to 1.5 times the mean ridge height. The algorithm starts searching at the top, in case multiple stable layers are present, the vertical potential temperature gradient is used to search for the stable layers. The vertical profile of the potential temperature gradient is computationally determined using the potential temperature derivative with respect to height (using a finite difference approach for each vertical layer). A ‘stable’ potential temperature gradient threshold was determined as *G* = 6 K km^−1^ by comparing the qualitatively defined CAPs in Section 2.2 to quantitatively defined CAPs determined by varying *G* between 3 and 10 K km^−1^. Two of the three case study winters (2015/2016 and 2021/2022) were used for this comparison and the results of this testing and are shown in Figure S1. If no point in the profile meets this threshold, then no integration height is defined, and therefore no CAP is present. There is high variability in the optimal values of *G* (between 5 and 10 K km^−1^) depending on the location and vertical resolution of the data. Using higher *G* values may be more appropriate for radiosonde profiles than for the ERA profiles because of the increased vertical resolution of the radiosonde data.

#### Step 2: Calculate Normalized VHD

Integration height significantly influences calculated *VHD* values. While changing the integration height improves the CAP classification by including stable layers above the ridge height, it also makes it more difficult to compare *VHD* values across days and locations, especially between locations with different integration heights. For example, a low integration height can result in low *VHD* values even when there is a strong, stable layer present. Similarly, high integration heights can lead to high *VHD* even when there is no stable layer. To address this issue, the normalized *VHD* method from Colgan et al. (2021) [6] is used. The *VHD* is normalized by calculating the ratio between the *VHD* calculated from the observed or modeled potential temperature profile and the *VHD* calculated using the potential temperature profile assuming an atmosphere that has a standard lapse rate (SLR), defined as 6.5 K km^−1^ [19], shown in Equation (2) as

(2) $${VHD}_{\mathrm{norm}}=\frac{VHD}{VHD_{SLR}}.$$

here, $VHD_{\mathrm{norm}}$ greater than one implies an average temperature profile that is more stable than the standard lapse rate of 6.5 K km^−1^. In Section S1, details about the interpretation of this normalization and how the *VHD* values can be used to compare bulk atmospheric stability across different days are provided.

#### Step 3: Calculate CAP VHD Thresholds

After calculating ${VHD}_{\mathrm{norm}}$ the next step is to determine a ${VHD}_{\mathrm{norm}}$ threshold that provides an indicator for CAP classification. Using a ${VHD}_{\mathrm{norm}}$ threshold of one, where an environmental profile is similar to the SLR, is not restrictive enough to classify CAPs [6]. To determine ${VHD}_{\mathrm{norm}}$ CAP threshold values, an iterative approach is used where the CAP classification calculations described above in Steps 1 and 2 are performed while adjusting the ${VHD}_{\mathrm{norm}}$ values used to determine the CAP threshold. This process was performed iteratively by comparing the CAP classification results from the algorithm using the ${VHD}_{\mathrm{norm}}$ threshold with the qualitative CAP evaluation test dataset for three winters (2010/2011, 2015/2016, and 2021/2022). The ${VHD}_{\mathrm{norm}}$ values with the greatest agreement, while also meeting the wind speed threshold (*WS* ≤ 4 m s^−1^), were selected as the CAP threshold value for each location.

#### Step 4: Automated CAP Classification

The final step is to use the ${VHD}_{\mathrm{norm}}$ threshold values to classify CAPs. For each vertical temperature profile a ${VHD}_{\mathrm{norm}}$ value is calculated and then compared with the threshold ${VHD}_{\mathrm{norm}}$ values, found in Step 3, for that location. If the calculated ${VHD}_{\mathrm{norm}}$ is greater than the threshold value and the surface wind speed is less than 4 m s^−1^, the profile is classified as a CAP. For vertical temperature profiles that meet the bulk atmospheric stability criteria, but not the surface wind speed threshold, a classification of ‘maybe’ is used, similar to the approach in Section 2.2. If both the stability criteria and wind speed threshold are not met it is considered a non-CAP. There are large uncertainties in modeled surface wind speeds; therefore, surface wind speed observations were used to apply the wind speed threshold for all datasets (radiosonde, ERA, and ERA_Adj_) in all locations.

## 3. Results

The results are shown starting with the location-specific ${VHD}_{\mathrm{norm}}$ threshold values to determine a CAP, comparing both the radiosonde and model vertical profiles. Then, results from three case studies follow to evaluate both the CAP classification method and using ERA model outputs for CAP classification. Finally, a summary of the number of CAP and PCAP events, at the locations shown in Figure 1, is provided for 2000–2022. The results of the ERA model evaluation are provided in Section S2.

### 3.1. VHD_norm_ CAP Threshold Values

After calculating ${VHD}_{\mathrm{norm}}$ (Steps 1 and 2 described in Section 2.3 above) the next step is to determine a ${VHD}_{\mathrm{norm}}$ threshold that provides an indicator for CAP classification (Step 3 in Section 2.3). Using data from three winters (corresponding to the case study winters), the ${VHD}_{\mathrm{norm}}$ threshold for each location is determined (Table 2). The CAP thresholds using the radiosonde observations are less than the thresholds when using ERA or ERA_Adj_. Threshold differences are minor between locations, though notably the Denver radiosonde threshold is lower than the rest, likely due to geographical differences (i.e., there is no contained valley in Denver, Table 1). ERA_Adj_, on average, has slightly lower thresholds than ERA, likely due to capturing more surface inversions that ERA misses. ERA_Adj_ is not computed in Denver because the surface station with the lowest elevation (Table 1) is above the surface elevation of the ERA grid.

### 3.2. Case Study 1: Winter 2010/2011

To evaluate the new automated CAP classification method the numerical results were compared with the qualitative evaluation test dataset, based on visual inspection, while also excluding the CAP cases with wind speeds exceeding the surface wind speed threshold. The results of this comparison are shown in Table 3. This table shows the agreement (as a percentage) of the visually inspected CAP and non-CAP days to the numerical CAP classification results. In all locations, there is a 78% or better agreement using vertical profiles from three different sources (radiosonde, ERA, and ERA_Adj_). The results based on radiosondes and ERA_Adj_ are generally similar, with the notable exception of the radiosonde-derived values in Denver. One downside of using the same potential temperature gradient threshold (*G*) for both ERA and radiosondes is that it can have better agreement with one over the other. In this case, the value of *G* that was selected is a better fit when using the ERA model than when using the radiosonde data in Denver. Comparing ERA and ERA_Adj_, the ERA_Adj_ improves the percentage of CAPs identified in Boise and Medford. However, in two cities (Reno and Salt Lake City), ERA_Adj_ performs slightly worse compared to the unadjusted ERA. In Medford, the ERA_Adj_ increases the efficacy from 78% to 92%, which is a significant improvement. This difference indicates that ERA does not adequately simulate the atmospheric surface layer on the valley floor in Medford. This could be due, in part, to the horizontal resolution of ERA, because the Rogue River Valley is relatively small (i.e., <30 km across) and the 31 km horizontal resolution of ERA is too coarse to capture the terrain.

The impact of the surface wind speed criteria on CAP classification is an important factor in the new classification method. For this test case, 6% of the profiles would be classified differently without the wind speed criteria (i.e., would be classified as a CAP). However, on average, the agreement compared to the visually determined CAPs is similar. Using the wind speed criteria in the CAP classification had the largest difference in Denver. The agreement between the numerical method and radiosonde visual inspection decreased by 12% when the wind speed criteria were not used.

Visually comparing the vertical temperature and wind speed profiles from radiosonde observations and ERA_Adj_ provides more context for the results shown in Table 3. While the majority of the time the new CAP classification method is suitable to use with profiles from both the radiosonde and ERA, there are cases when the new CAP determination method works with the radiosonde data, but not with ERA or ERA_Adj_. The discrepancies in the CAP classification method will be investigated further using example temperature profiles.

Figure 2a shows a plot from Medford on 5 January 2011. ${VHD}_{\mathrm{norm}}$ in this example is 2.78 using ERA_Adj_ and 4.66 using the radiosonde, and both are well above the ${VHD}_{\mathrm{norm}}$ thresholds in Table 2 (1.41 and 1.23, respectively), indicating a CAP. The *VHD* integration heights for both the radiosonde and ERA_Adj_ are higher than the visualized inversion height in both examples. Also, the integration heights for both profiles are above the mean ridge height because there is a stable layer (with *dθ*/*dz* > *G* = 6 K km^−1^) above the temperature inversion layer. The significant temperature change at the surface in the ERA_Adj_ temperature profile comes from appending the surface station data at the bottom of the model temperature profile. While not plotted, because above the surface ERA and ERA_Adj_ profiles are identical, ERA has a substantially weaker surface inversion than is indicated by the ERA_Adj_ profile (i.e., the surface temperature in ERA is greater than the temperature from the surface meteorological station).

Figure 2b shows an example of an elevated temperature inversion in Reno on 4 January 2011. Note that both the radiosonde and ERA_Adj_ have the same surface temperature value because both vertical temperature profiles have surface observations appended from the valley floor in Reno. ${VHD}_{\mathrm{norm}}$ is 2.60 using ERA_Adj_ and 2.29 using the radiosonde. Both values are above the ${VHD}_{\mathrm{norm}}$ threshold for Reno, as shown in Table 2 (1.33 and 1.03 for ERA_Adj_ and radiosonde, respectively) and are considered CAPs. Additionally, the impacts of the coarse vertical resolution of ERA are evident in this figure, where the strength of the inversion is smoothed out in the ERA vertical temperature profile, but ERA still captures a stable layer at the same height.

Figure 2c shows a plot from Salt Lake City on 28 November 2010. ${VHD}_{\mathrm{norm}}$ using ERA_Adj_ is 2.32 and 7.49 using the radiosonde. This is above the ${VHD}_{\mathrm{norm}}$ threshold in Table 2 (1.55 and 1.19, respectively) and both are classified as CAPs, but the difference in ${VHD}_{\mathrm{norm}}$ is large. Visually, the difference is shown by a strong, stable layer above the surface in the radiosonde observations, while the ERA_Adj_ profile indicates a surface inversion. ERA_Adj_, having the adjusted surface temperature observation, modifies the ERA model temperature profile so that it is labeled as a CAP. The integration height for ERA_Adj_ is much higher than the radiosonde integration height, which decreases ${VHD}_{\mathrm{norm}}$ because the area above the surface inversion in ERA is only slightly stable. This is a case where the ERA model does not capture a strongly elevated inversion.

Using results from the Persistent Cold Air Pool Study (PCAPS [20]), conducted during this winter in Salt Lake City, additional comparisons can be made. PCAPS has more vertical profile data than the twice-daily radiosondes from the airport, providing a more cohesive dataset to compare with the new CAP classification method. PCAPS also includes data that was not ingested in the ERA data assimilation process. Another test of our new CAP classification method is to compare the days we classify as CAPs during PCAPS to the CAP days identified by the PCAPS investigators. Our new CAP classification method using ERA and ERA_Adj_ captures 93% of the CAP days, while using the radiosonde captures 98%. It should be noted that the PCAPS CAP days are not necessarily the IOP days of the study but only the days where CAPs were observed. The failure of ERA to find every CAP day in the PCAPS study highlights a limitation of using coarse horizontal and vertical resolution model data, as some CAPs are missed because of model smoothing or uncertainties in the modeling the atmospheric surface layer in ERA.

### 3.3. Case Study 2: Winter 2015/2016

Similarly to the previous section, radiosonde profiles during winter 2015/2016 were visually inspected and categorized into CAP and non-CAP days if there was a stable atmospheric layer observed in the temperature profiles. The results from the qualitative, visual inspection were compared to the new CAP classification method, where the agreement between the two methods is shown in Table 4. The ERA_Adj_ method performs similar, or slightly worse, to the ERA method in all cities, except Medford. However, on average, the radiosondes, ERA, and ERA_Adj_ all perform about the same.

Figure 3a shows a plot from Salt Lake City on 23 January 2016. The radiosonde observations show a stable layer to about the mean ridge height, with two inversion layers above the surface, one starting at ~1.5 km ASL and a second inversion layer starting at ~2.0 km ASL. The integration height for the radiosonde profile captures both inversion layers. ERA smooths out these features and shows a nearly isothermal layer from the surface to ~3.0 km ASL. ${VHD}_{\mathrm{norm}}$ using the radiosonde is 2.32, well above the ${VHD}_{\mathrm{norm}}$ threshold in Table 2 (1.19). Using ERA_Adj_, ${VHD}_{\mathrm{norm}}$ is 2.18 which is also above the threshold of 1.55. The observed surface wind (approximately 5 m s^−1^) is above the threshold of 4 m s^−1^, which classifies both the ERA_Adj_ and radiosonde profiles as a non-CAP. This is an example of a CAP day where the wind speed threshold is too strict and is classified as a non-CAP.

Figure 3b shows a plot from Boise on 31 December 2015. ${VHD}_{\mathrm{norm}}$ is 1.53 using ERA_Adj_ and 1.74 using the radiosonde. Both thresholds are above the thresholds outlined in Table 2 (1.40 and 1.23 using ERA_Adj_ and the radiosonde, respectively). The radiosonde profile shows a temperature inversion, while ERA shows a slightly stable layer and does not capture the temperature inversion. Regardless of these differences, using our new classification method this case is successfully labeled as a CAP.

Figure 3c shows data from Medford on 4 February 2016. ${VHD}_{\mathrm{norm}}$ is 1.49 using ERA_Adj_ and 2.19 using the radiosonde, so this example is classified as a CAP. The profiles have significant differences, however. The observations show a shallow elevated temperature inversion layer at ~1 km ASL, with a thin unstable layer at the surface. While ERA_Adj_ does not have an inversion layer, there is an elevated slightly stable layer starting at ~1 km ASL. If only data below the mean ridge height are considered, ${VHD}_{\mathrm{norm}}$ is 1.08 using ERA_Adj_, indicating that the layer is only slightly more stable than the SLR.

### 3.4. Case Study 3: Winter 2021/2022

Like the previous examples, the winter 2021/2022 radiosondes were visually inspected to classify each day as a CAP or non-CAP day. This qualitative, visual inspection dataset was compared to the results from the new CAP determination method. Table 5 shows the agreement between days visually determined CAPs and non-CAPs to the automated CAP determination method. The agreement between the CAP determination method and the visually determined CAP and non-CAP days ranges from 84 to 98% for ERA and 87–98% for ERA_Adj_, and 88–92% for the radiosonde observations. ERA_Adj_ performs better in Boise and Medford, while the unadjusted ERA model performs better in Las Vegas. However, results from ERA and ERA_Adj_ are similar. Medford again stands out where ERA_Adj_ performs better than ERA for CAP classification, which is likely due to the horizontal resolution of the ERA model in that region. The CAP determination using the radiosonde performs better or is equivalent to ERA or ERA_Adj_ in Salt Lake City, Boise, Las Vegas, and Denver.In contrast, CAP determination using the ERA model performs better in Reno (ERA and ERA_Adj_ similar) and Medford (ERA_Adj_).

Figure 4a shows a plot from Boise on 18 December 2021. ${VHD}_{\mathrm{norm}}$ using ERA_Adj_ is 1.84 and 2.08 using the radiosonde. ${VHD}_{\mathrm{norm}}$ using ERA_Adj_ is above the CAP threshold listed in Table 2 (1.40) and above the threshold (1.23) for the radiosonde. In the ERA_Adj_ profile, there is an isothermal stable layer from ~2–3 km ASL. Approximately half of this isothermal layer extends above 1.5 times the mean ridge height, the height limit where the new CAP classification method stops searching for stable layers. However, because the bottom half of this elevated stable layer is below the maximum integration height it is captured in the ${VHD}_{\mathrm{norm}}$ calculation and is why it is greater than one. This is an example of a CAP case that would be missed if only the layer below the mean ridge height was considered.

Figure 4b shows an elevated, relatively shallow, temperature inversion in the vertical temperature profile from Reno on 21 January 2022. ${VHD}_{\mathrm{norm}}$ using ERA_Adj_ is 2.46 and 3.23 using the radiosonde. Both values are above the ${VHD}_{\mathrm{norm}}$ threshold in Table 2 (1.33 and 1.03, respectively), and both are labeled CAPs. ERA_Adj_ matches the shape of the vertical profile from radiosonde observations, and also captures the shallow elevated inversion layer at the top of the CAP. The integration height found for both the radiosonde and ERA_Adj_ are similar and correctly identifies the top of the stable layer. The CAP classification method correctly designates each profile as a CAP. This example also illustrates how using a method with variable integration height, which can go above the mean ridge height, can improve *VHD* calculations.

Figure 4c shows the temperature profile in Las Vegas on 28 November 2021. ${VHD}_{\mathrm{norm}}$ using ERA_Adj_ is undefined as no point in the profile satisfies the potential temperature gradient criteria *G* = 6 K km^−1^ and therefore no integration height is found for the *VHD* calculation. ${VHD}_{\mathrm{norm}}$ using the radiosonde is 1.60, which is above the CAP threshold of 1.08 from Table 2. The observed and ERA_Adj_ modeled temperature profiles are similar. However, there are several small isothermal layers in the radiosonde temperature profiles that are not captured in the ERA_Adj_ temperature profile. Had ${VHD}_{\mathrm{norm}}$ using ERA_Adj_ been calculated to the mean ridge height, ${VHD}_{\mathrm{norm}}$ would be 1.42, which meets the threshold in Table 2 (1.18). This is an example of the set value of G that is used to search for the integration height in the temperature profile failing to capture a CAP.

### 3.5. CAP and PCAP Summary Statistics

One objective of this work is to numerically quantify CAP days using large datasets, and to apply a new CAP classification method to regions without radiosonde observations. This section provides a summary of how many days in during winter were classified as CAPs. Additionally, discussion of how the results compare when using radiosonde observations and ERA model results is also given. Examining patterns between similar regions and year-to-year differences provides some insight into overall CAP formation and the effectiveness of the new CAP classification method, especially between using the ERA model and observations. Based on the test case results shown above, the radiosonde observations typically have the best agreement with manually determined CAPs over three winters, compared to ERA, where the number of CAP days is underestimated.

Figure 5 shows the number of CAP days per year for each location using radiosonde observations, ERA, and ERA_Adj_. There is a common trend among most cities, where more CAPs are found using the radiosonde data compared to using ERA outputs. The two main reasons for this are vertical resolution differences, where ERA has coarser vertical resolution, and uncertainties in the ERA model with modeling the atmospheric surface layer.

When comparing the CAPs found using the Salt Lake City radiosonde data and CAPs found using ERA_Adj_ in Ogden and Provo, Salt Lake City averages 44 CAPs per year, Ogden averages 47 per year, and Provo averages 44 per year. Ogden is in the same basin as Salt Lake City, so it is notable that, on average, slightly more CAPs are found there. Typically, ERA underestimates the number of CAP events, and in Ogden using ERA_Adj_ has more CAPs than in Salt Lake City using radiosonde data. Investigating this further, model error could be a factor in this discrepancy because radiosonde data are assimilated into the ERA model, therefore, ERA should be most reliable near Salt Lake City (i.e., radiosonde launch location). However, this does not seem to be the case because both ERA and ERA_Adj_ results in Salt Lake City show average yearly CAP days as 36 and 35, respectively. This is on average approximately 10 fewer CAPs per year than in Ogden and Provo using both ERA and ERA_Adj_, and in Salt Lake City using the radiosonde. Essentially, the Ogden and Provo ERA results match the CAP classification numbers from Salt Lake City radiosonde observations better than the Salt Lake City ERA results. Vertical resolution differences help explain the discrepancy between the radiosonde and ERA results, but they do not explain the differences between Salt Lake City, Ogden, and Provo. These differences could potentially come from the coarse horizontal resolution of the ERA model along the Wasatch Front.

Elsewhere, Denver has the lowest number of CAPs because it is not contained in a valley. Medford has the largest difference in CAP classifications between the ERA model results and the radiosonde, similar to the case study results discussed above. In Medford, the radiosonde finds a CAP, on average, for two-thirds of the winter days, which is mainly due to the deep, narrow Rogue River Valley where Medford is located. As mentioned above, the ERA model has high uncertainties in Medford because of the coarse horizontal resolution, so the large differences between the ERA and the radiosonde results are expected.

There are no radiosonde observations in the Central Valley, but based on the CAP classifications using the ERA model, the Central Valley locations have the most CAP days on average. There are often periods where the stable marine layer is active in a part of the valley, which results in more CAP days. There is some variability among the locations within the valley, but generally, the year-to-year trend is similar among all locations.

To classify PCAPs, Whiteman et al. (2014) [2] proposed a method based on the *VHD* being greater than a CAP threshold value for more than 36 h. In this study, because we are only using the afternoon soundings, and we assume the morning sounding will also indicate a CAP, we classify a PCAP when there are two or more CAP days in a row. Knowing the number of PCAP days each year and investigating the PCAP climatology, including the number of PCAP versus CAP days for each location, can be insightful. For example, a year with a relatively dry winter could potentially have many PCAP events, as extended high pressure subsidence will increase the likelihood of PCAP events. Conversely, storms break up PCAPs resulting in fewer events, but snow cover can lead to stronger PCAP events.

The number of PCAP days by year in each location using radiosonde observations, ERA, and ERA_Adj_ is shown in Figure S3. Similarly to the CAP results, the region-specific PCAP effects are apparent. In Salt Lake City, Reno, Boise, Las Vegas, Medford, Ogden, and Provo, the number of PCAP days follow a similar pattern to the number of CAP days. In Denver, there are fewer PCAP days compared to other locations, except during winter 2006–2007 and winter 2015/2016. Snow cover enhances CAP persistence due to the increased surface albedo [21]. In Denver, based on snowfall data from the National Weather Service (NWS), 138.7 cm (54.6 in) of snowfall was recorded between 15 November 2006 and 15 February 2007. This is the second largest snowfall amount recorded over a 15 November to 15 February period since records began. Winters 2011–2012 and 2011–2012 also had significant snowfall in Denver, both of those winters also had notable increases in observed (i.e., radiosonde) CAP and PCAP days.

Another factor associated with an increase in the number of PCAP days, or increased PCAP length, is decreased precipitation, fewer storms, or drought. Drought in the western U.S. is associated with wintertime high-pressure ridges that block cyclonic activity [22]. This blocking results in decreased storm activity and increased high-pressure subsidence that can lead to stable ABLs, resulting in CAPs with fewer opportunities to break up multi-day CAP events (i.e., PCAPs). This may explain the increase in PCAP days in Reno and Medford in winter 2013–2014. According to data from the NWS, this winter was notably dry with Reno reporting 50% of average precipitation on 15 February 2014, while Medford had received 25% of normal precipitation until early February. Fewer storms led to fewer opportunities for PCAPs to break up.

In all locations, there is significant variability in the number of PCAP days each winter. The year-to-year variability is beyond the scope of this investigation, but winters with fewer PCAPs could be associated with warmer temperatures, stormy weather, less snow cover, or cloudy CAPs, where several processes across atmospheric scales influence CAP formation and duration. There are more PCAP days using radiosondes versus ERA, as expected based on the results presented above where ERA underestimates the number of CAP events.

## 4. Discussion

Numerical CAP determination is difficult because there is not a clear, uniform quantitative definition of a CAP. We implemented a numerical CAP classification method based on comparing the numerical results with a qualitative analysis of CAPs over three winters. The qualitative analysis was performed by visual inspection of vertical profiles, defining a CAP when a stable layer was found in the vertical temperature profile. The numerical method focused on testing a quantitative definition that could find a similar pattern for CAPs in large datasets (i.e., multiple locations over decades). The difference between this new CAP classification method and previous methods (e.g., Whiteman et al. (2014) [2] and Colgan et al. (2021) [6]) is using a variable integration height for calculating *VHD*, slight modifications from Colgan et al. (2021) [6] for calculating ${VHD}_{\mathrm{norm}}$, and using ERA model data in locations without radiosonde observations.

Each city has different topography which affects CAP formation and dissipation, which is reflected in the average number of CAPs per year shown in Section 3.5. Complex vertical boundary layer structures in temperature, moisture, and wind complicate CAP classification methods. California’s Central Valley between two mountain ranges is often affected by subsidence inversions above the inland penetration of the marine layer, which results in a high number of CAP days per year.

Our CAP classification approach focuses on vertical profiles of observed, or modeled analyses, of potential temperature relative to what might be expected assuming a SLR potential temperature profile extending upwards from the surface temperature through a depth that is location dependent. This approach captures CAP intensity well when a strong, stable layer lies somewhere below the location-dependent layer depth (i.e., between the surface and 1.5 times the mean ridge height). High ${VHD}_{\mathrm{norm}}$ values larger than one indicate that the observed (radiosonde) or analyses (ERA) profiles contain less moisture and are more stable than the SLR profile.

The examples shown in Section S1 illustrate some limitations in our numerical CAP classification method. For example, we assume a dry (i.e., unsaturated) atmospheric layer and are not accounting for moisture that can impact stability through latent heat exchange. This means for our method the SLR represents a stable atmospheric layer, where for a saturated layer the SLR would only be weakly stable. Also, the potential temperature gradient used to select the integration height (*G* = 6 K km^−1^) corresponds to a temperature profile with a lapse rate less than the moist adiabatic lapse rate. This criteria requires a strong, stable layer to be present in the environmental temperature profile for our numerical method to find an integration height and calculate VHD, *VHD*_*SLR*_, and ${VHD}_{\mathrm{norm}}$.

Our CAP classification method is intended to be useful in locations without observed vertical profiles (i.e., locations without radiosonde observations). The inability of the ERA analyses to capture low near-surface temperatures in valleys and basins during CAPs may lead to underestimating the number or intensity of CAPs in those regions. This issue is evident in Medford, where the use of the ERA model captures significantly fewer CAP events than when radiosonde observations are used. In addition, the reduced vertical resolution of the model analyses may also lead to underestimating CAP intensity.

This study examined only 00 UTC vertical profiles; therefore, the diurnal CAP formation, maintenance, and dissipation are not examined in this study. Overnight and early morning surface- or near-surface stable layers are common. It is also possible for a CAP to break up during the day and be re-established by 00 UTC. Using hourly ERA or other model analyses would improve the CAP classification method by providing better diagnosis of the temporal evolution of the boundary layer structure.

The adjusted ERA method used here requires an observed near-surface temperature observation that might not be available for every model grid cell. However, our results show that using ERA without the surface station can still be useful for CAP classification.

A benefit of this new CAP classification method is that it includes stable layers aloft. During PCAP events, the capping stable layer is not always near the surface and is sometimes well above the mountain ridge height. Our method considers these events as CAP days. Other methods that only consider the profiles below the mean ridge height will miss these conditions and potentially mischaracterize a CAP. While air quality will typically be better for these types of CAPs compared to a surface inversion as they may allow for near-surface vertical mixing, the stable layer aloft traps pollutants and is important to consider.

The CAP classification method presented here is relatively simple and could be improved to perform better when using model analysis or forecast profiles. One improvement would be to adjust the method to determine integration height as a function of model vertical resolution. In addition, while we used three case study winters to determine location-specific ${VHD}_{\mathrm{norm}}$ thresholds (Table 2), future work could simplify this approach by using a standardized ${VHD}_{\mathrm{norm}}$ threshold applied for all locations. Future work could also use other models to simulate or classify CAPs. For example, using High Resolution Rapid Refresh/Gridded Forecast System analyses at 3 km horizontal resolution, or a fine-resolution WRF could resolve some of the horizontal and vertical resolution problems limiting the use of the ERA. Considering these additional improvements, the CAP classification method could be modified for greater agreement to what is observed by visual inspection for the CAP strength.

## 5. Summary

A CAP classification method was developed to compare CAPs in cities with and without radiosondes. The new method includes elevated CAPs that are above the mean ridge height, which were missed with previous approaches that use the mean ridge height for *VHD* integration. The CAP classification method was optimized to sufficiently capture CAPs found by radiosondes and using a relatively coarse numerical model (ERA), despite vertical resolution differences. Adjustments were made for ERA (ERA_Adj_) to replace the modeled surface temperature with an observed temperature, as the lowest level of the valley may not always be adequately simulated. A similar adjustment was made for the Reno radiosonde data because the radiosonde is launched above the valley floor, i.e., an observation from the valley floor was appended to the bottom of the radiosonde vertical profile.

A three-winter test case was used to evaluate the CAP determination method and showed agreement on 80–95% days when using radiosonde observations at all locations. ERA generally has good agreement with radiosonde observations for temperature, wind speed, and *VHD*. The average surface temperature bias over the entire time period is approximately 1 °C. The average ERA surface wind speed bias is about 4 m s^−1^, which is significant and can influence the CAP classification. Adjusting the surface wind speed from ERA using observations roughly halves this bias. The surface wind speed threshold in the CAP classification method used the observed valley-floor wind speed for both radiosondes and ERA because of this bias. The *VHD* values calculated using ERA are lower than the values derived from the radiosonde because thin elevated stable layers are smoothed out in ERA. ${VHD}_{\mathrm{norm}}$ values calculated from ERA temperature profiles are often greater than the values derived from radiosondes for similar reasons. The CAP classifications based on ERA profiles, on average, underestimate the number of CAPs when compared to observations. Identification of CAPs using ERA_Adj_ has slightly worse agreement compared to the CAP classifications using radiosondes, but is more reliable than ERA, on average. Considering the large difference in vertical resolution between the radiosonde observations and ERA, the uncertainties in CAP classification using ERA are reasonable. However, in certain regions, especially in Medford where the model horizontal resolution is too coarse, ERA does not capture the terrain sufficiently for CAP classification.

## Supplementary Material

SI

**Supplementary Materials:** The following supporting information can be downloaded at: https://www.mdpi.com/article/10.3390/atmos16121325/s1, Section S1 Interpretation of the Normalized *VHD* and Section S2 Comparison of Model Results (ERA) to Observations (Radiosondes). Table S1 with the latitude and longitude of the observations, Tables S2–S4 with the ERA and observations comparison results, Figure S1 with results from the potential temperature gradient threshold value testing, Figure S2 showing the idealized vertical profiles for the interpretation of the Normalized *VHD*, and Figure S3 with the number of PCAP days in each location.

## Figures and Tables

**Figure 1.**
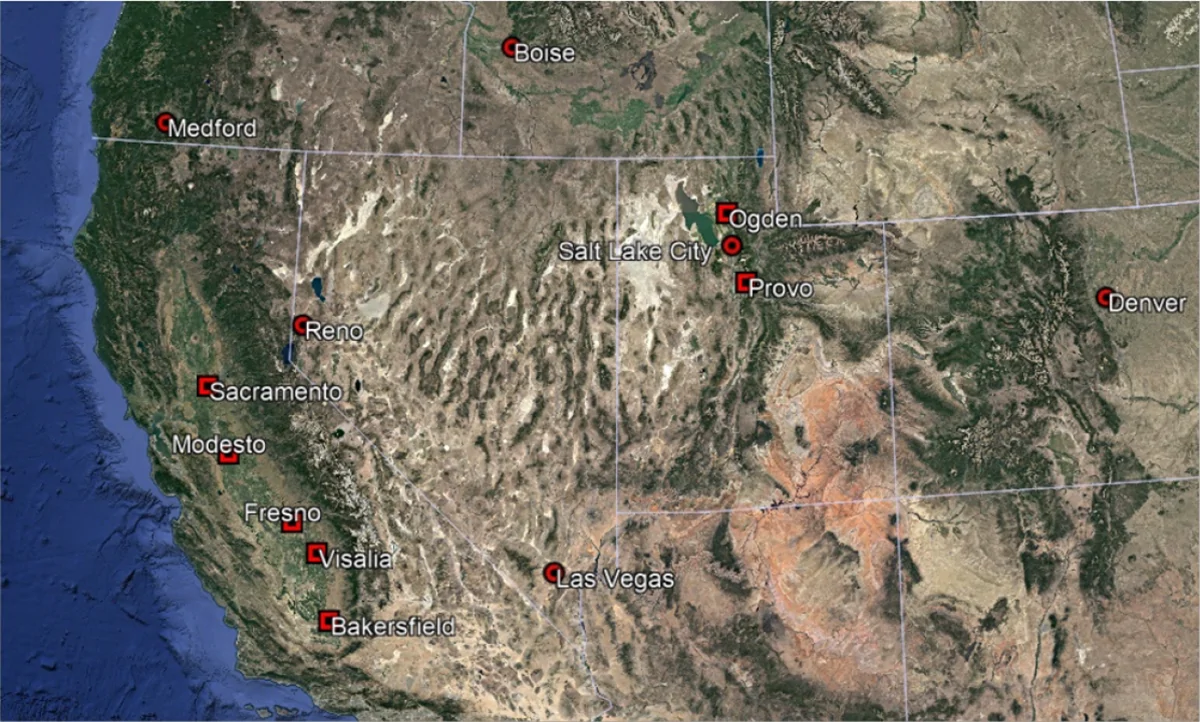
Map of the locations in this study that use meteorological reanalysis models to quantify CAP events. Cities with radiosonde data available to evaluate the model results are indicated with circles. *Note: The latitude and longitude of the locations are provided in* Table S1.

**Figure 2.**
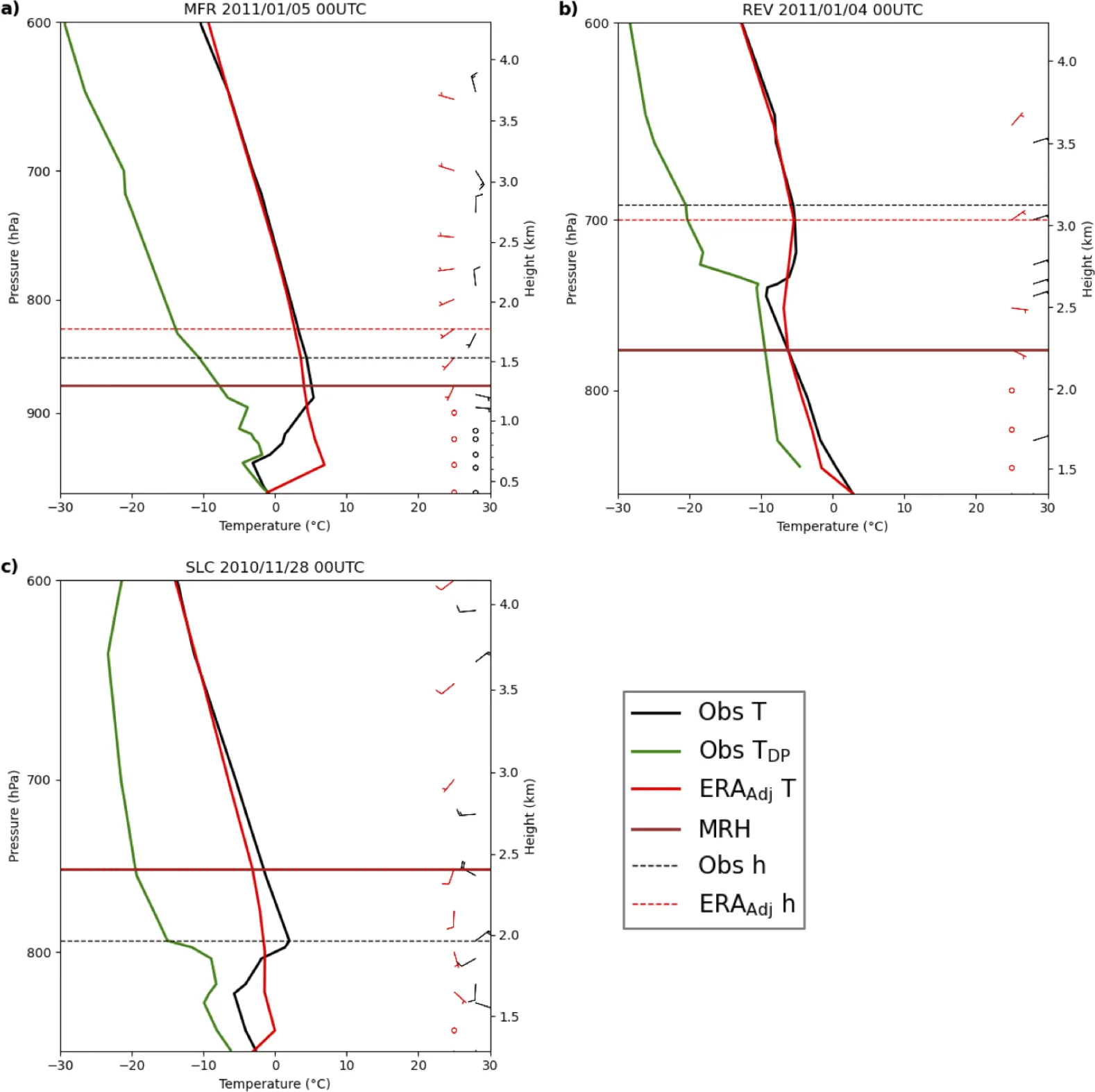
Vertical temperature and wind profile plots from winter 2010/2011 showing temperature and wind profiles of ERA (red), radiosonde temperature and wind (black), radiosonde dew point temperature (green), mean ridge height (MRH–dark red), integration height (*h*) from radiosonde profile (dotted black), and integration height (*h*) from ERA (dotted red). Shows an example from (**a**) Medford, (**b**) Reno, and (**c**) Salt Lake City.

**Figure 3.**
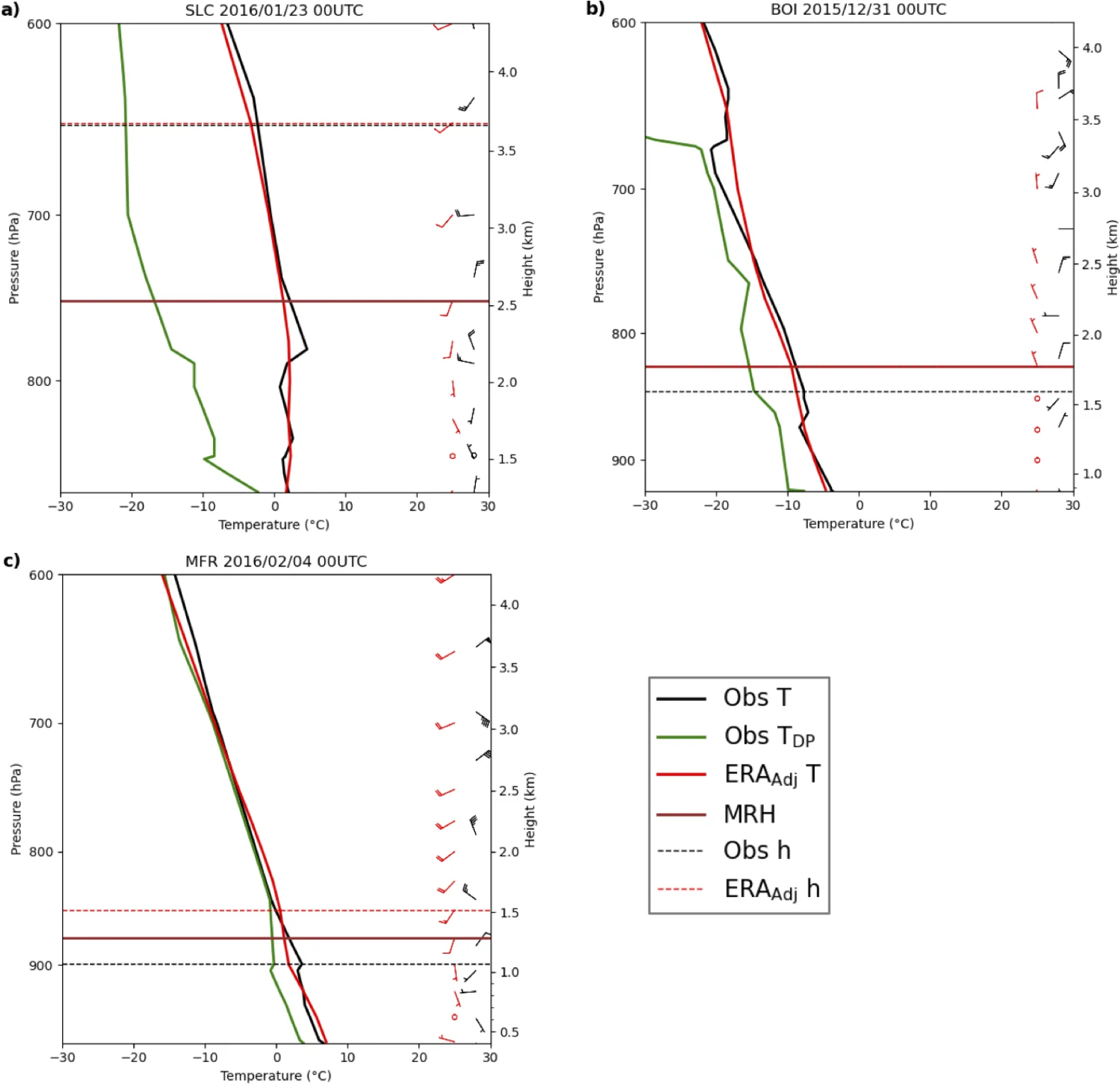
Vertical temperature and wind profile plots from winter 2015/2016 showing temperature and wind profiles of ERA (red), radiosonde temperature and wind (black), radiosonde dew point temperature (green), mean ridge height (MRH–dark red), integration height (*h*) from radiosonde profile (dotted black), and integration height (*h*) from ERA (dotted red). Shows an example from (**a**) Salt Lake City, (**b**) Boise, and (**c**) Medford.

**Figure 4.**
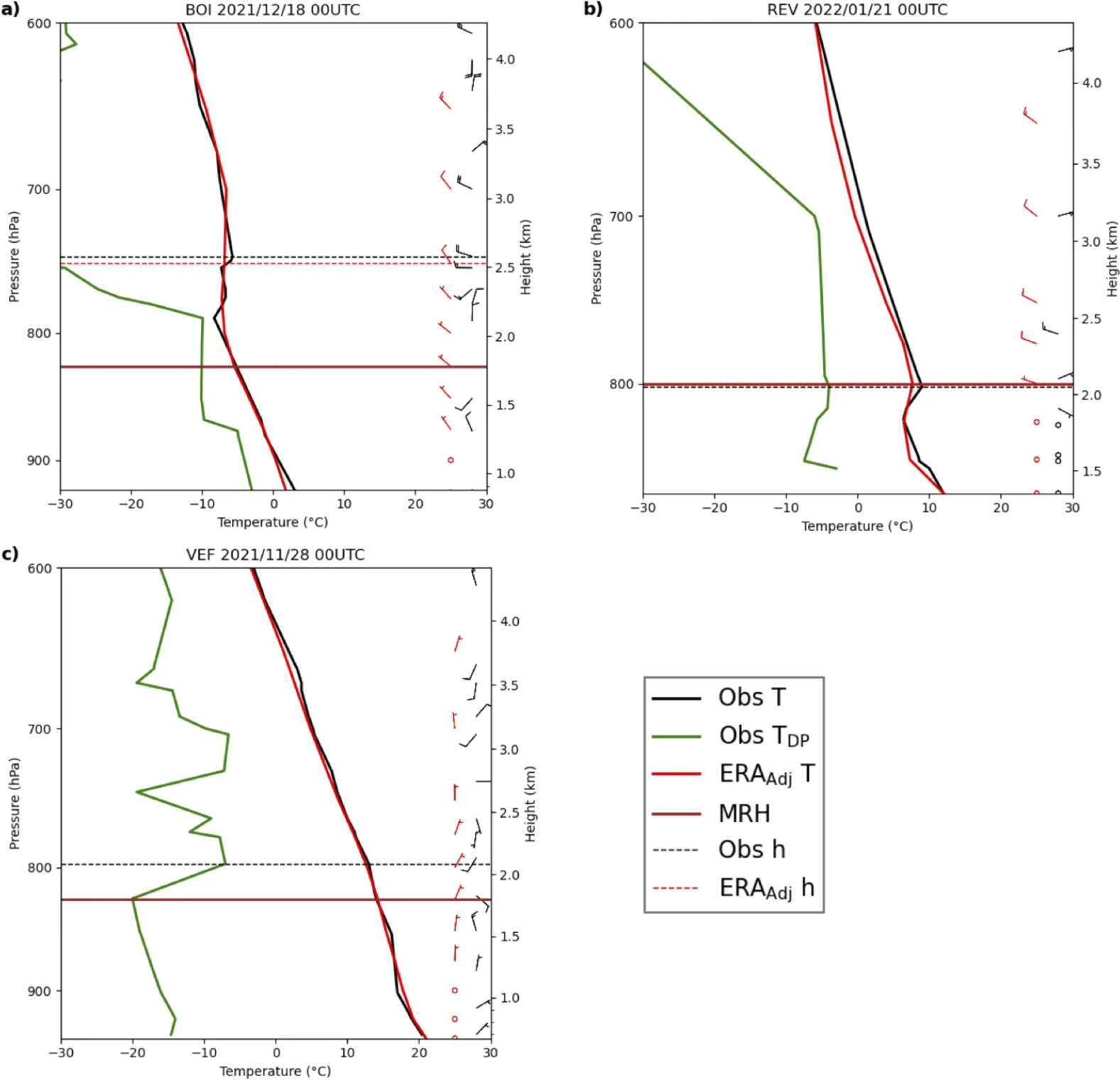
Vertical temperature and wind profile plots from winter 2021/2022 showing temperature and wind profiles of ERA (red), radiosonde temperature and wind (black), radiosonde dew point temperature (green), mean ridge height (MRH-dark red), integration height (*h*) from radiosonde profile (dotted black), and integration height (*h*) from ERA (dotted red). Shows an example from (**a**) Boise, (**b**) Reno, and (**c**) Las Vegas.

**Figure 5.**
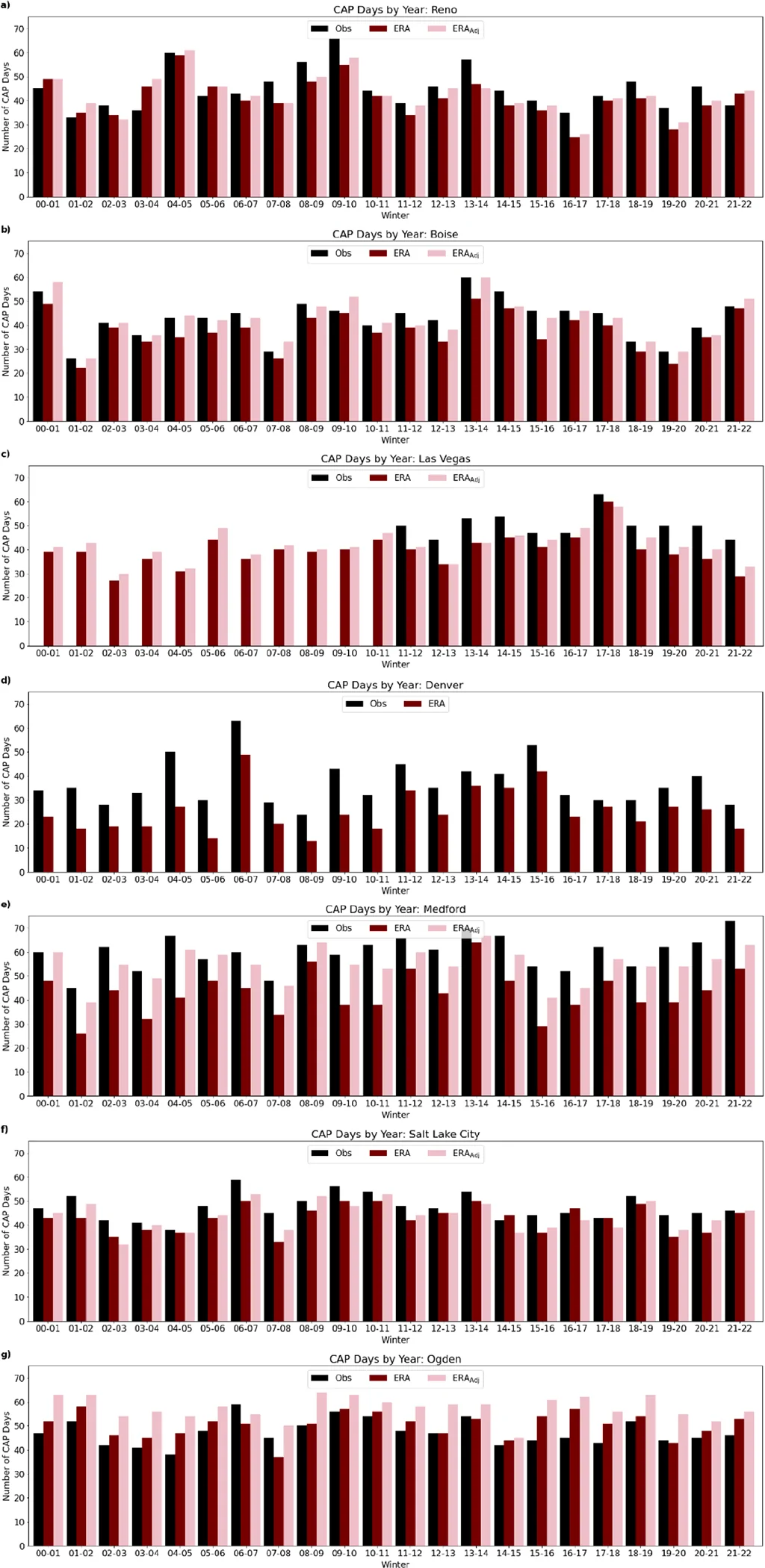

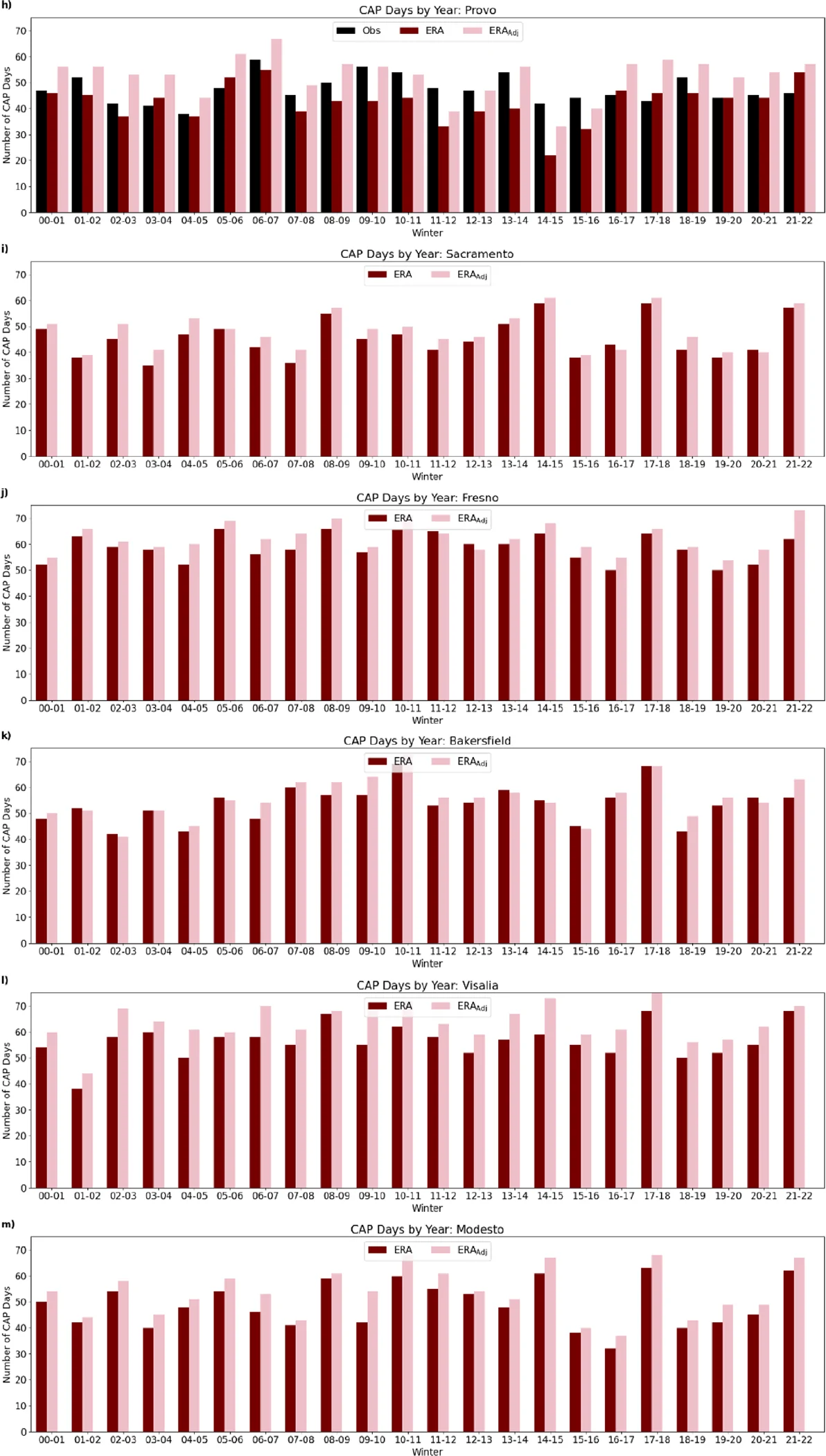
Number of CAP days in each location for each winter from 2000/2001 through 2021/2022, based on radiosonde data (black), ERA (red), ERA_Adj_ (pink). (**a**) Reno, (**b**) Boise, (**c**) Las Vegas, (**d**) Denver, (**e**) Medford, (**f**) Salt Lake City, (**g**) Ogden, (**h**) Provo, (**i**) Sacramento, (**j**) Fresno, (**k**) Bakersfield, (**l**) Visalia, and (**m**) Modesto. *Note: The Ogden and Provo radiosonde observations are from the Salt Lake City radiosonde.*

**Table 1.** Elevation and basin characteristics for each location, including radiosonde and ASOS surface meteorological station information.

City	Basin L x W (km)	Mean Ridge Height (m ASL)	Surface Met Station	Surface Elevation (m ASL)	ERA Surface Elevation (m ASL)	Radiosonde Station	Radiosonde Launch Elevation (m ASL)
Bakersfield	700 x 90	1501	KBFL	155	195	-	-
Boise	100 x 100	1829	KBOI	860	867	BOI	874
Denver	-	2757	KBKF	1700	1684	DNR	1625
Fresno	700 x 125	1844	KFAT	101	193	-	-
Las Vegas	50 x 30	1708	KLAS	664	848	VEF	697
Medford	45 x 25	1219	KMFR	400	405	MFR	405
Modesto	700 x 90	1433	KMOD	26	194	-	-
Ogden	50 x 40	2269	KOGD	1353	1540	-	-
Provo	45 x 20	2560	KPVU	1371	1540	-	-
Reno	20 x 30	2134	KRNO	1342	1542	REV	1516
Sacramento	700 x 90	1189	KSMF	6	197	-	-
Salt Lake City	50 x 30	2438	KSLC	1288	1313	SLC	1288
Visalia	700 x 120	1453	KVIS	90	192	-	-

Note: Surface elevation is the same elevation as the surface met station, except for Denver where the surface met station is higher (1700 m ASL) than the radiosonde. Also, because Denver is not contained within a valley, basin dimensions are not given.

**Table 2.** Threshold values of *VHD_norm_* to identify CAPs using the automated CAP classification method for each city, where a *VHD_norm_* value greater than the value in this table indicates a CAP.

City	Radiosonde	ERA	ERA_Adj_
Salt Lake	1.19	1.54	1.55
Reno	1.03	1.54	1.33
Boise	1.23	1.51	1.4
Las Vegas	1.08	1.33	1.18
Denver	0.83	1.37	-
Medford	1.23	1.43	1.41
Ogden	-	1.64	1.42
Provo	-	1.52	1.47
Sacramento	-	1.74	1.56
Fresno	-	1.51	1.43
Bakersfield	-	1.45	1.43
Visalia	-	1.57	1.39
Modesto	-	1.65	1.49

**Table 3.** Percentage agreement between visually determined CAP and non-CAP days compared to the automated numerical CAP classification method during winter 2010/2011. Results are shown for radiosonde observations, ERA, and ERA_Adj_ at each location, including the average across all locations.

Winter 2010/2011			
City	Radiosonde	ERA	ERA_Adj_
Salt Lake City	93%	91%	88%
Reno	86%	86%	84%
Boise	94%	92%	96%
Denver	80%	95%	-
Medford	88%	78%	92%
**Average**	**88%**	**88%**	**90%**

**Table 4.** Percentage agreement between visually determined CAP and non-CAP days compared to the automated numerical CAP classification method during winter 2015/2016. Results are shown for radiosonde observations, ERA, and ERA_Adj_ at each location, including the average across all locations.

Winter 2015/2016			
City	Radiosonde	ERA	ERA_Adj_
Salt Lake City	89%	87%	85%
Reno	85%	89%	85%
Boise	89%	90%	84%
Las Vegas	95%	95%	94%
Denver	78%	83%	-
Medford	86%	75%	89%
**Average**	**87%**	**87%**	**87%**

**Table 5.** Percentage agreement between visually determined CAP and non-CAP days compared to the automated numerical CAP classification method during winter 2021/2022. Results are shown for radiosonde observations, ERA, and ERA_Adj_ at each location, including the overall average of each method across all locations.

Winter 2021/2022			
City	Radiosonde	ERA	ERA_Adj_
Salt Lake City	92%	98%	98%
Reno	89%	93%	93%
Boise	89%	86%	89%
Las Vegas	92%	88%	87%
Denver	92%	92%	-
Medford	88%	84%	87%
**Average**	**90%**	**90%**	**91%**

## Data Availability

Radiosonde data can be accessed from the Atmospheric Soundings–Wyoming Weather Web website (https://weather.uwyo.edu/upperair/sounding.html, (accessed on 18 November 2025)). Automated surface observing system (ASOS) surface weather station data can be accessed from MesoWest (https://mesowest.utah.edu/, (accessed on 18 November 2025)). The European Centre for Medium-Range Weather Forecasts (ECMWF) Reanalysis v5 (ERA5) can be assed from the Copernicus Climate Data Store (https://cds.climate.copernicus.eu/datasets/reanalysis-era5-single-levels?tab=overview, (accessed on 18 November 2025)).

## Abbreviations

The following abbreviations are used in this manuscript:

ABL: Atmospheric boundary layer
ASOS: Automated surface observing system
CAP: Cold-air pool
DALR: Dry adiabatic lapse rate
ECMWF: European Centre for Medium-Range Weather Forecasts
ERA: European Centre for Medium-Range Weather Forecasts Reanalysis v5
MALR: Moist adiabatic lapse rate
MetUM: Met Unified Model
MRH: Mean ridge height
NAAQS: National Ambient Air Quality Standard
NARR: North American Regional Reanalysis
NWS: National Weather Service
PCAP: Persistent cold air pool
PCAPS: Persistent Cold Air Pool Study
SLR: Standard lapse rate
VHD: Valley heat deficit
WRF: Weather Research and Forecasting Model
