# Supplementary material for: Leveraging Meteorological Reanalysis Models to Characterize Wintertime Cold Air Pool Events Across the Western United States from 2000 to 2022
Source: Atmosphere (Basel). Author manuscript; Available in PMC 2026 Nov 11. (PMC13750838; doi:10.3390/atmos16121325)
Supplement: Supplementary file 1 [file NIHMS2217888-supplement-SI.pdf]

Supplementary Materials:

Jacob Boomsma[1] and Heather A. Holmes[2,*]

Table S1. Latitude and longitude of surface meteorology stations and radiosonde launch sites.

City	ASOS Coordinates	Radiosonde Coordinates
Bakersfield	35.43, -119.06	
Boise	43.57, -116.24	43.57, -116.22
Denver	39.71, -104.76	39.77, -104.88
Fresno	36.78, -119.72	
Las Vegas	36.07, -115.16	36.05, -115.18
Medford	42.38, -122.88	42.37, -122.87
Modesto	37.63, -120.95	
Ogden	41.19, -112.02	
Provo	40.22, -111.72	
Reno	39.51, -119.77	39.57, -119.80
Sacramento	38.70, -121.59	
Salt Lake City	40.77, -111.97	40.77, -111.97
Visalia	36.32, -119.40	

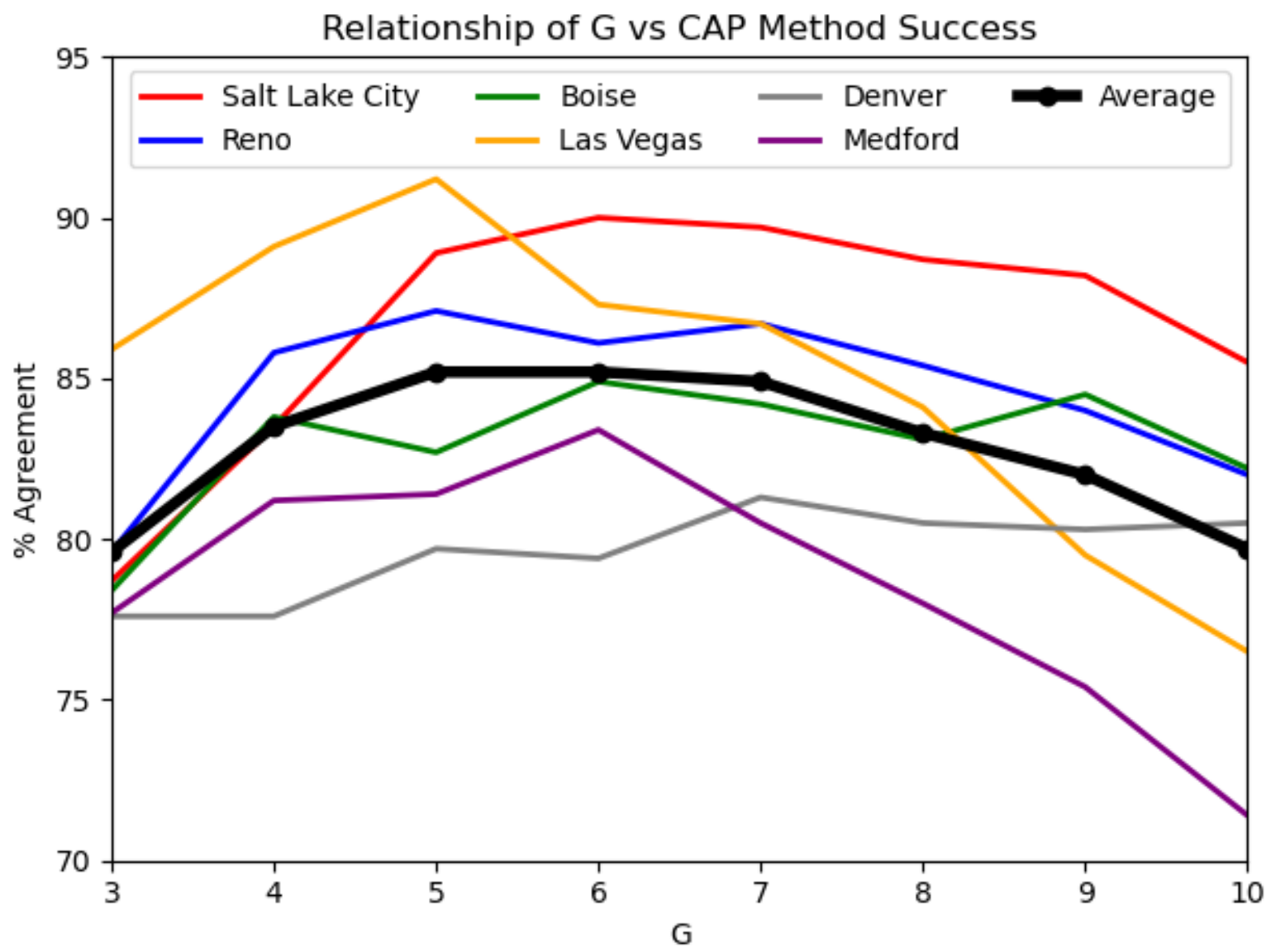

Figure S1. Results for two winters (2015/2016 and 2021/2022) comparing the potential temperature gradient threshold values (G) and CAP agreement in locations with radiosonde observations (color lines) and average across all locations (black line).

S1. Interpretation of the Normalized VHD

In our CAP classification method, VHD_{norm} is used to classify CAPs; therefore, it is important to understand the scenarios that lead to a high or low VHD_{norm} value. Figure S2 illustrates examples of vertical temperature and potential temperature profiles using idealized, but still common, examples of wintertime temperature profiles. The red and black lines indicate the temperature and potential temperature profiles for the idealized environmental and standard lapse rates, and the corresponding shaded areas are proportional to the VHD and VHD_{SLR} values, respectively. VHD_{norm} would be proportional to the red shaded area (VHD) divided by the black shaded area (VHD_{SLR}). The variable integration height, h, is also shown for each example. For the following examples, an unsaturated atmospheric layer is assumed to avoid the complexities associated with moisture and latent heat exchange, which impacts atmospheric stability.

Figure S2a shows an example temperature and potential temperature profile for an idealized shallow (~300 m deep) surface-based temperature inversion, compared to the SLR of 6.5 K km-1. For a dry atmospheric layer, the SLR (black lines) indicates a stable atmosphere, and therefore, there is a small positive VHD_{SLR} associated with the profile. The idealized lapse rate (red lines) indicates a surface inversion, a strongly stable layer, with a positive VHD value larger than VHD_{SLR}. In this example, because the VHD value for the inversion layer is greater than the VHD_{SLR} for the same vertical layer, it results in a large VHD_{norm}.

An example where there is both a surface and an elevated inversion is shown in Figure S1b. While the height in Figure S2b is limited to the lowest kilometer of the atmosphere, this example would still be relevant if the elevated inversion was above the mean ridge height. Using an integration height above both inversion layers, rather than an integration height just above the surface inversion, increases VHD and VHD_{SLR} compared to the surface inversion example in Figure S2a. VHD_{norm} decreases slightly compared to the shallow surface inversion, but still indicates a stable atmospheric layer.

The third example in Figure S2c shows an idealized elevated temperature inversion. VHD is much larger than the VHD_{SLR}, which results in VHD_{norm} being greater than 1. However, VHD_{norm} is lower than in the previous example, as there is only an elevated inversion and no surface inversion. While the integration height in this idealized example would likely be below the mean ridge height, the results using our numerical CAP classification method would be similar if the elevated inversion was above the mean ridge height (i.e., our method allows for integration height up to 1.5 times the mean ridge height).

Figure S2d shows an isothermal layer from the surface up to 1 km, which is a stable layer and should be classified as a CAP. The integration height in this case is 1 km, resulting in a large (> 1) value for VHD_{SLR}. Because the entire layer is stable up to the integration height, the VHD value is the largest of all four examples shown in Figure S2. The value of VHD_{norm} is similar to the value associated with the example of the idealized elevated temperature inversion (Figure S2c), even though the VHD for the isothermal layer is ~1.5 MJ m^{-2} higher.

For cases when the environmental lapse rate is greater than or equal to the DALR, the integration height is undefined (i.e., no stable layer is found). When no integration height is found, VHD_{norm} cannot be calculated. However, if we use an integration height based on the maximum vertical extent of the atmosphere considered (1.5 times the mean ridge height), then VHD would be a negative value, resulting in a negative VHD_{norm}. We classify all profiles with $VHD_{norm} < 0$ as non-CAP, and remove the numerical value of VHD_{norm} because it is considered undefined.

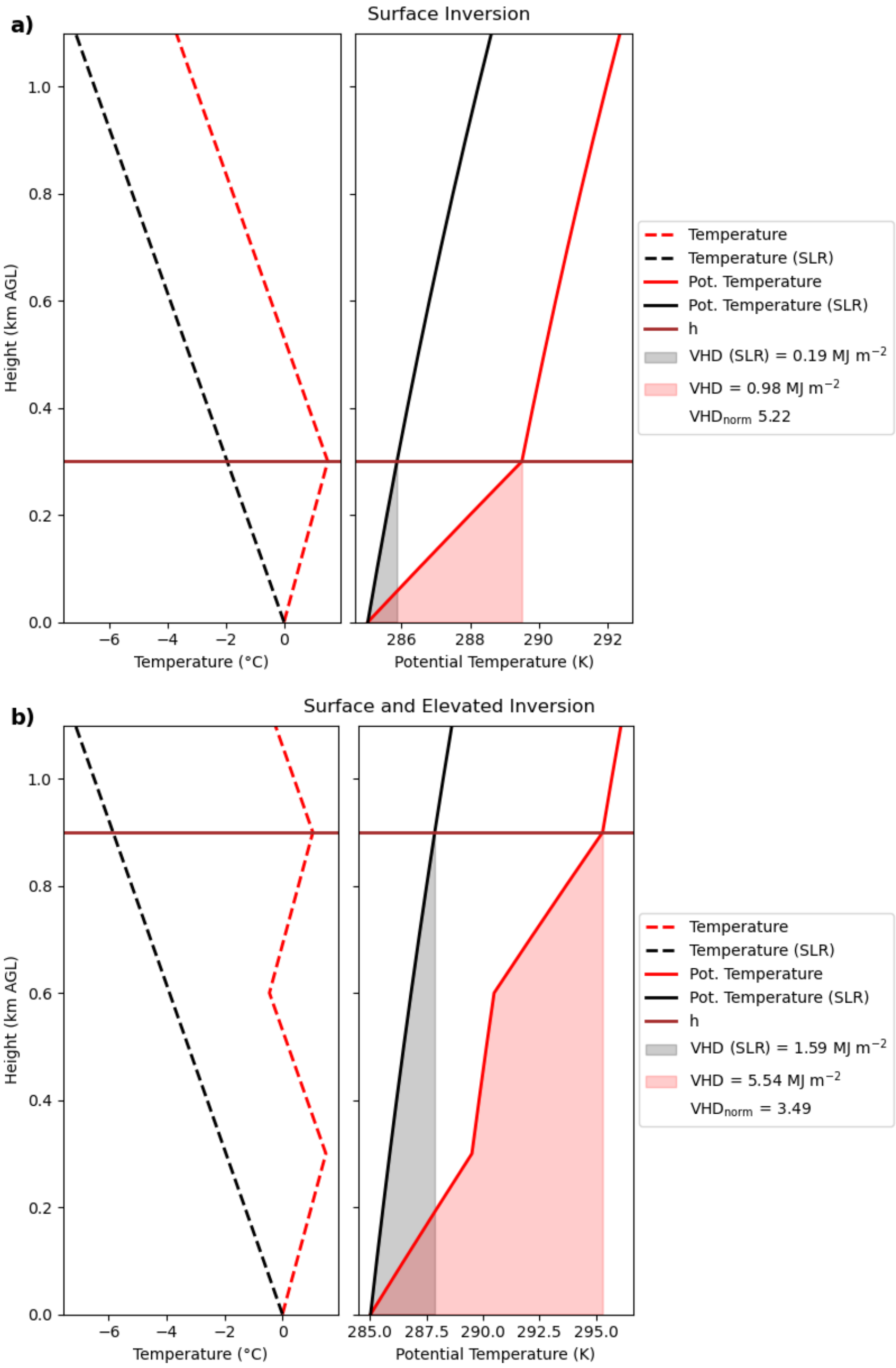

Figure S2. continued.

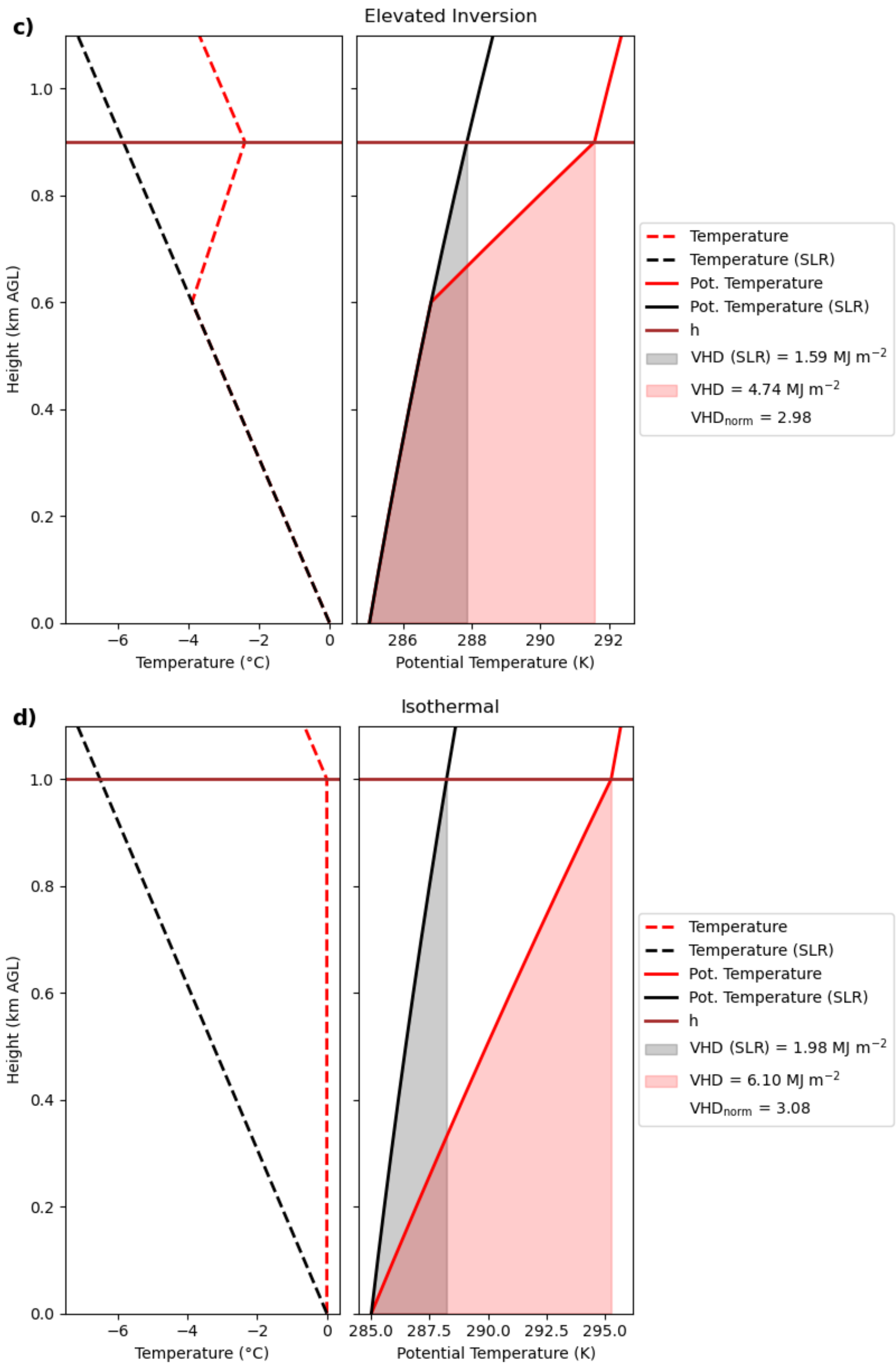

Figure S2. Examples of idealized potential temperature profiles representing environmental lapse rates for different atmospheric conditions. Figures on the right side show potential temperature profiles for comparison. The VHD for each profile is proportional to the area shaded. In order, the images show (a) idealized surface inversion, (b) surface and elevated inversion, (c) elevated inversion, and (d) isothermal layer.

S2. Comparison of Model Results (ERA) to Observations (Radiosondes)

An important consideration in using the ERA model output for CAP classification, necessary for locations without radiosondes, is the effectiveness of ERA to simulate the vertical temperature and wind speed profiles. The model evaluation is performed for ERA and ERA_{Adj}, where ERA_{Adj} is the vertical profiles from the ERA where surface station data replaces the surface point provided by ERA. The model outputs are compared to radiosonde measurements when available. Understanding the model error compared to observations is necessary to ensure that models can reasonably substitute radiosondes.

There are a few considerations to note before looking into the results. First, because this is a reanalysis product, radiosondes are assimilated into the model. Therefore, ERA will perform better in regions with radiosondes than without. Additionally, when analyzing ERA_{Adj} surface values, the bias is not zero because there are often subtle differences between the lowest point of the radiosonde dataset and the reading at the nearest surface station. This could be due to subtle changes in the environment, instrumentation differences, or marginal timing discrepancies. For example, ERA_{Adj} in Reno uses surface observations from KRNO, roughly 7 km away and 174 m lower in elevation than the radiosonde launch site in Reno, while other stations are closer geographically.

Table S2 shows the evaluation statistics for the temperature profiles of the radiosonde observations, ERA, and ERA_{Adj} for the 22-year period. It is important to note that the ERA surface heights (shown in Table 1) may not be representative of the actual surface. The surface temperature bias is relatively low, with a maximum bias of 1.7 °C in Reno. The vertical profile from the radiosonde does not reach the valley floor in Reno; therefore, the radiosonde surface value is taken at a higher elevation, and a greater surface temperature bias is expected.

Temperature bias for the 750 hPa and 500 hPa pressure levels is, on average, less than 1°C, with the bias decreasing with increasing elevation. This is expected because model errors associated with CAPs involve the surface or near-surface conditions[14]. The correlation coefficient between the balloon and ERA and ERA_{adj} temperature is high ($R=0.99$ in all locations for both models). The ERA temperature root mean squared error (RMSE) ranges from 2.5 °C to 2.74 °C. The RMSE and bias increase for ERA_{Adj} but remain low, with an RMSE of less than 4.94 °C and a bias of less than 2.9 °C.

Table S3 shows the wind speed statistics over the entire 22-year period in each location. Wind speed evaluation metrics are more variable across the locations than temperature. Part of this is a timing issue. While the surface observations and balloon launch times are the same, or within a few minutes, balloon observations in the upper atmosphere are later than the launch time. Additionally, replacing the surface point from ERA with surface station observations reduces the bias in surface wind speed considerably on average (in all locations except Boise). ERA, on average, underestimates the surface wind speed in every location.

The mean bias of the wind speed does not decrease with height, as evidenced by the wind speed bias at the 750 hPa and 500 hPa pressure levels. The mean bias is highest at the 500 hPa level in three locations. Considering the full vertical profile of wind speed, the correlation values are still high, with the lowest being 0.74 in Medford and the highest being 0.85 in Reno. Comparing the correlations for the ERA and ERA_{Adj} wind speed (0.74-0.85 and 0.74-0.84, respectively) with the balloon, the correlation is similar in all locations. The RMSE values for wind speed are lower in each location for ERA_{Adj} (9.5-10.1 m s^{-1}) compared to ERA (9.7- 10.5 m s^{-1}). The mean bias of the entire profile is low in both ERA and ERA_{Adj}, and there is no major difference between the two.

Table S4 shows the average VHD, integration height (h), VHD_{norm}, and number of CAP events using the new CAP classification method for the entire 22-year period. Comparing the ERA and ERA_{Adj} model outputs to the balloon output, the average VHD is much larger when derived from the balloon data in all cases except Reno. One explanation is the value of h, where the VHD integration height using radiosonde observations is higher in Salt Lake City, Reno, Denver, and Boise. Higher h values will correspond to a higher VHD, assuming a general increase in potential temperature with height. Another factor is the vertical resolution of the observed profile being fine enough that variability in the temperature profile can be resolved. Higher VHD values are calculated when using radiosonde data because thin stable layers are resolved in the observations while being smoothed out in ERA. The highest VHD_{norm} from radiosonde observations is in Medford, and the lowest VHD_{norm} is in Reno. This is expected as Medford is in a deep, sheltered valley, and Reno is a shallower, windier valley. The number of CAP days in all locations except Salt Lake City increases when using ERA_{Adj} compared to ERA. In all locations, using the radiosonde data results in more CAP

days than using ERA. The increased resolution of radiosonde observations allows for more CAPs to be identified, as thinner stable layers can be captured.

Table S2. Temperature statistics for 22 winters of afternoon vertical profiles, comparing the radiosonde (Obs), ERA, and ERA_{Adj}. The surface, 750 hPa, 500 hPa, and full profiles are compared in each city. *Note: The upper-level comparisons are the same for ERA and ERA_{Adj}, indicated by '-' and R, RMSE, and T_{Full} are calculated from the surface to 500 hPa.*

	Obs	*ERA*			*ERA_{Adj}*		
City	Mean $T_{surface}$	Mean $T_{surface}$	Mean Bias $T_{surface}$		Mean $T_{surface}$	Mean Bias $T_{surface}$	
Salt Lake City	2.2°C	1.9°C	-0.3°C		2.7°C	0.5°C	
Reno	6.4°C	4.7°C	-1.7°C		7.9°C	1.5°C	
Boise	3.1°C	3.4°C	0.4°C		3.8°C	0.7°C	
Denver	4.8°C	3.8°C	-1.0°C		4.4°C	-	
Medford	7.5°C	9.0°C	1.5°C		8.6°C	1.1°C	
City	Mean T_{750}	Mean T_{750}	Mean Bias T_{750}		Mean T_{750}	Mean Bias T_{750}	
Salt Lake City	-2.9°C	-3.4°C	-0.5°C		-	-	
Reno	0.0°C	-0.4°C	-0.4°C		-	-	
Boise	-3.9°C	-3.8°C	0.1°C		-	-	
Denver	0.3°C	-0.3°C	-0.6°C		-	-	
Medford	-1.4°C	-1.6°C	-0.1°C		-	-	
City	Mean T_{500}	Mean T_{500}	Mean Bias T_{500}		Mean T_{500}	Mean Bias T_{500}	
Salt Lake City	-21.0°C	-21.0°C	0.0°C		-	-	
Reno	-18.9°C	-19.0°C	-0.1°C		-	-	
Boise	-21.9°C	-21.7°C	0.2°C		-	-	
Denver	-20.8°C	-20.9°C	0.0°C		-	-	
Medford	-20.1°C	-20.2°C	-0.1°C		-	-	
City		R	RMSE	Bias T_{Full}	R	RMSE	Bias T_{Full}
Salt Lake City		0.99	2.50°C	0.0°C	0.99	4.03°C	2.0°C
Reno		0.99	2.74°C	-0.2°C	0.99	4.94°C	2.9°C
Boise		0.99	2.50°C	0.3°C	0.99	4.31°C	2.5°C
Denver		0.99	2.57°C	-0.1°C	-	-	-
Medford		0.99	2.52°C	0.0°C	0.99	3.84°C	1.9°C

Table S3. Wind speed statistics for 22 winters of afternoon vertical profiles, comparing the radiosonde (Obs), ERA, and ERA_{Adj}. The surface, 750 hPa, 500 hPa, and full profiles are compared in each city. *Note: The upper-level comparisons are the same for ERA and ERA_{Adj}, indicated by '-' and R, RMSE, and T_{Full} are calculated from the surface to 500 hPa.*

	Obs	*ERA*			*ERA_{Adj}*		
City	Mean $WS_{surface}$	Mean $WS_{surface}$	Mean Bias $WS_{surface}$		Mean $WS_{surface}$	Mean Bias $WS_{surface}$	
Salt Lake City	5.3 m s^{-1}	2.2 m s^{-1}	-3.1 m s^{-1}		6.7 m s^{-1}	2.2 m s^{-1}	
Reno	6.8 m s^{-1}	1.5 m s^{-1}	-5.3 m s^{-1}		5.8 m s^{-1}	-1.0 m s^{-1}	
Boise	4.8 m s^{-1}	3.9 m s^{-1}	-0.9 m s^{-1}		6.8 m s^{-1}	2.0 m s^{-1}	
Denver	7.5 m s^{-1}	3.7 m s^{-1}	-3.8 m s^{-1}		-	-	
Medford	7.6 m s^{-1}	1.1 m s^{-1}	-6.5 m s^{-1}		3.0 m s^{-1}	-4.6 m s^{-1}	
City	Mean WS_{750}	Mean WS_{750}	Mean Bias WS_{750}		Mean WS_{750}	Mean Bias WS_{750}	
Salt Lake City	22.4 m s^{-1}	20.0 m s^{-1}	-2.4 m s^{-1}		-	-	
Reno	21.2 m s^{-1}	21.4 m s^{-1}	0.2 m s^{-1}		-	-	
Boise	16.3 m s^{-1}	16.8 m s^{-1}	0.5 m s^{-1}		-	-	
Denver	26.7 m s^{-1}	24.0 m s^{-1}	-2.7 m s^{-1}		-	-	
Medford	7.2 m s^{-1}	14.7 m s^{-1}	7.5 m s^{-1}		-	-	
City	Mean WS_{500}	Mean WS_{500}	Mean Bias WS_{500}		Mean WS_{500}	Mean Bias WS_{500}	
Salt Lake City	6.2 m s^{-1}	7.9 m s^{-1}	1.7 m s^{-1}		-	-	
Reno	4.8 m s^{-1}	11.2 m s^{-1}	6.3 m s^{-1}		-	-	
Boise	4.6 m s^{-1}	8.6 m s^{-1}	3.9 m s^{-1}		-	-	
Denver	8.3 m s^{-1}	11.0 m s^{-1}	2.7 m s^{-1}		-	-	
Medford	4.8 m s^{-1}	8.6 m s^{-1}	3.7 m s^{-1}		-	-	
City		R	RMSE	Bias WS_{Full}	R	RMSE	Bias WS_{Full}
Salt Lake City		0.85	9.7 m s^{-1}	-1.1 m s^{-1}	0.84	9.5 m s^{-1}	-2.0 m s^{-1}
Reno		0.78	10.3 m s^{-1}	0.8 m s^{-1}	0.79	9.8 m s^{-1}	-0.5 m s^{-1}
Boise		0.83	9.9 m s^{-1}	0.4 m s^{-1}	0.82	9.7 m s^{-1}	-0.5 m s^{-1}
Denver		0.81	10.1 m s^{-1}	-1.3 m s^{-1}	-	-	-
Medford		0.74	10.5 m s^{-1}	1.9 m s^{-1}	0.74	10.1 m s^{-1}	1.0 m s^{-1}

Table S4. Average and standard deviation (s) for the stability characteristics and number of CAPs for 22 years, comparing the radiosondes (Obs), ERA, and ERA_{Adj}. VHD, VHD_{norm}, h, and CAP classifications are determined using the CAP classification method presented in this paper.

	Obs				ERA			
City	Mean VHD (MJ m^{-2})	σ VHD (MJ m^{-2})	h (m)	σ h (m)	Mean VHD (MJ m^{-2})	σ VHD (MJ m^{-2})	h (m)	σ h (m)
Salt Lake City	12.38	8.87	3164	632	10.05	10.52	3042	643
Reno	6.58	5.7	2805	486	4.88	5.61	2708	406
Boise	8.37	13.41	2374	521	7.09	7.58	2315	446
Denver	6.95	7.92	3063	832	4.47	7.56	3007	793
Medford	5.37	4.71	1445	470	3.57	3.81	1633	254
City	Mean VHD_{norm}	σ VHD_{norm}	# of CAPs		Mean VHD_{norm}	σ VHD_{norm}	# of CAPs	
Salt Lake City	1.96	1.2	565		2.3	0.72	525	
Reno	1.72	1.49	516		2.57	1.19	453	
Boise	2.32	1.47	527		2.44	0.9	458	
Denver	2.33	2.21	443		2.39	0.99	331	
Medford	2.61	1.74	748		2.1	0.6	536	

	ERA_{Adj}			
City	Mean VHD (MJ m^{-2})	σ VHD (MJ m^{-2})	h (m)	σ h (m)
Salt Lake City	10.93	10.94	2950	740
Reno	5.55	6.22	2664	440
Boise	10.61	8.3	2168	588
Medford	4.51	4.53	1444	470
City	Mean VHD_{norm}	σ VHD_{norm}	# of CAPs	
Salt Lake City	2.33	0.95	524	
Reno	2.28	1.15	471	
Boise	2.68	1.45	508	
Medford	2.69	2.06	664	

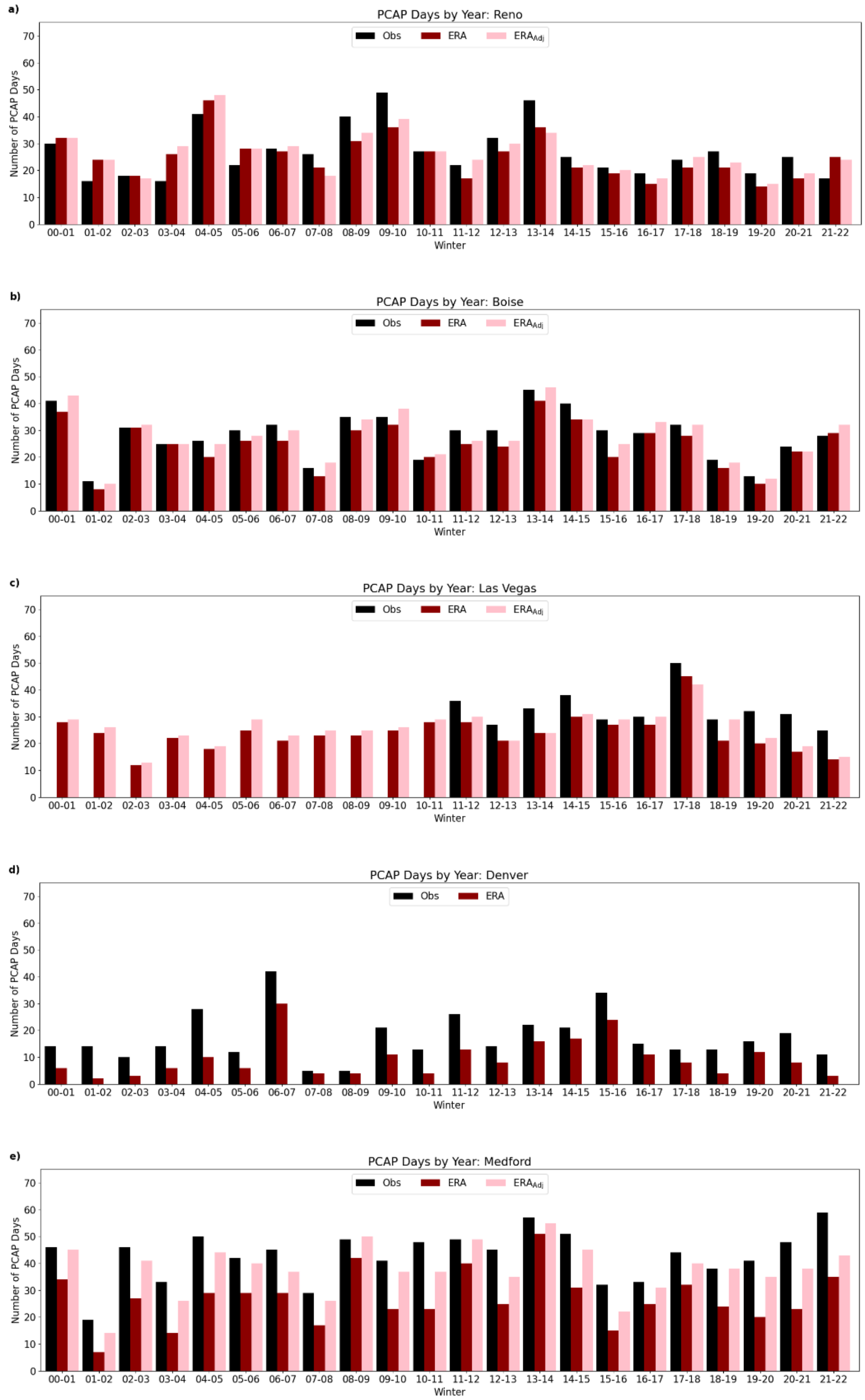

Figure S3. continued.

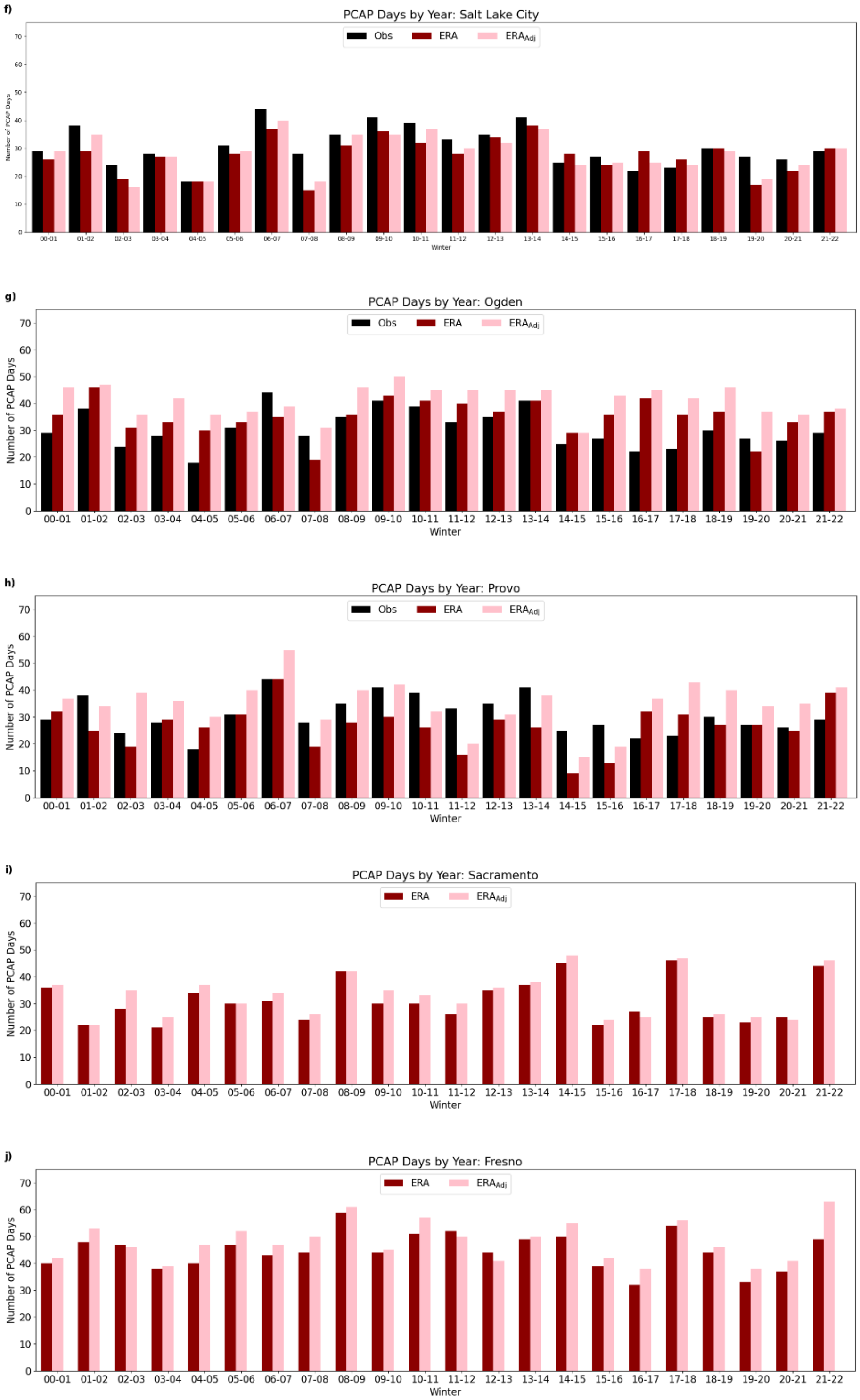

Figure S3. continued.

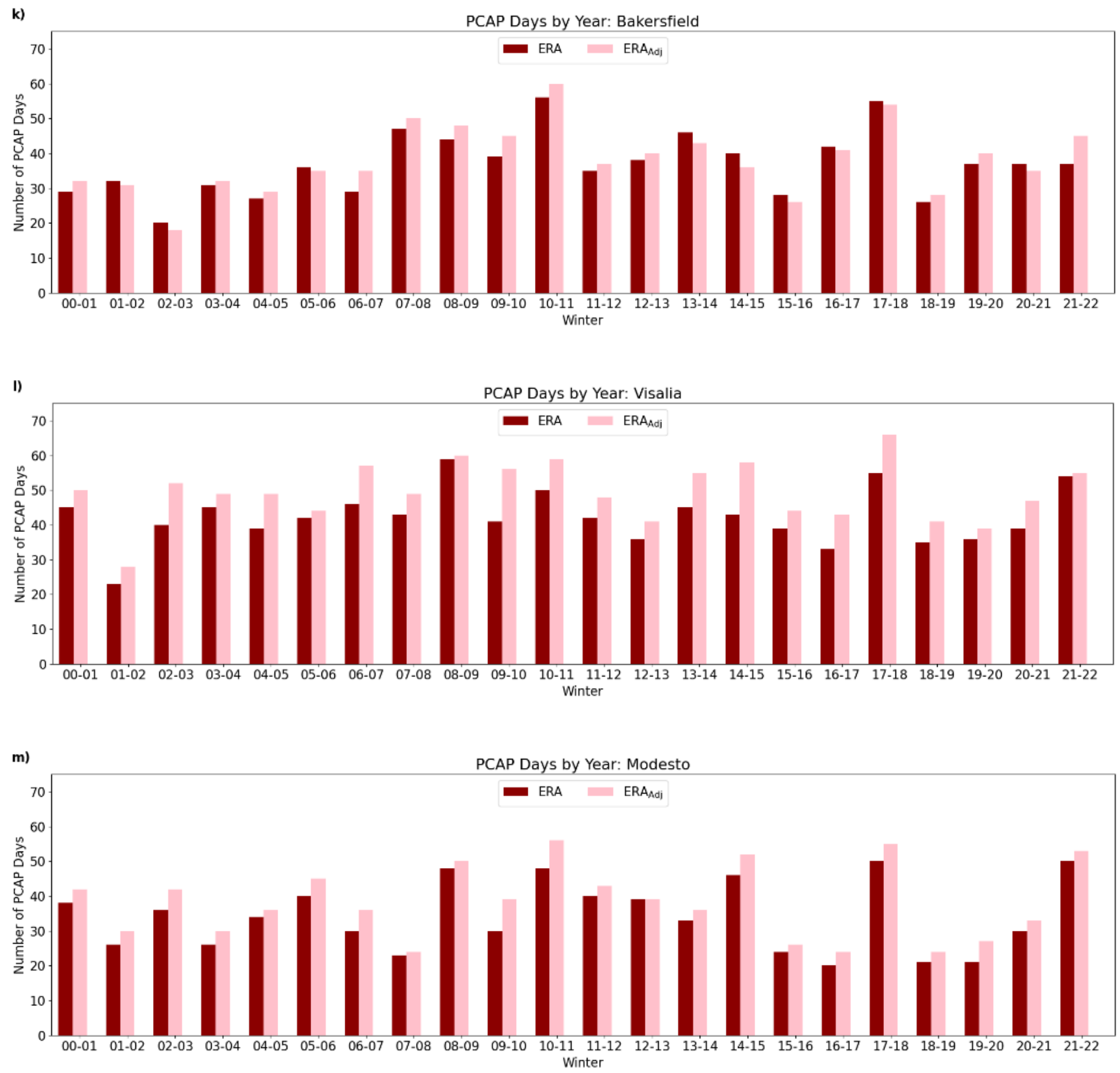

Figure S3. Number of PCAP days in each location for each winter from 2000/2001 through 2021/2022, based on radiosonde data (black), ERA (red), and ERA$_{Adj}$ (pink). (a) Reno, (b) Boise, (c) Las Vegas, (d) Denver, (e) Medford, (f) Salt Lake City, (g) Ogden, (h) Provo, (i) Sacramento, (j) Fresno, (k) Bakersfield, (l) Visalia, and (m) Modesto. The Ogden and Provo radiosonde observations are from the Salt Lake City radiosonde.
